# AMIGO2 is a pivotal therapeutic target related to M2 polarization of macrophages in pancreatic ductal adenocarcinoma

**DOI:** 10.18632/aging.205380

**Published:** 2024-01-05

**Authors:** Shensi Chen, Wujun Du, Ke Feng, Ke Liu, Cunji Li, Shengming Li, Hua Yin

**Affiliations:** 1 Department of Gastrointestinal Surgery, General Hospital of Ningxia Medical University, Yinchuan, Ningxia 750004, China; 2 Department of Emergency, General Hospital of Ningxia Medical University, Yinchuan, Ningxia 750004, China; 3 Department of Gastroenterology, General Hospital of Ningxia Medical University, Yinchuan, Ningxia 750004, China

**Keywords:** pancreatic cancer, M2 polarization, gene set enrichment analysis, machine learning, AMIGO2

## Abstract

Pancreatic ductal adenocarcinoma (PDAC) is a common kind of lethal cancer, with low early diagnostic rate and poor prognosis. In this study, we identified and verified the AMIGO2 with significant diagnostic and prognostic value in PDAC through LASSO regression combined with multiple machines learning methods, including RVM-RFE and Random Forest in TCGA and GEO datasets. The relevance between the expression of AMIGO2 and M2 polarization of macrophages was identified through pancancer, normal tissue, and cell lines data in TCGA, GTEx and CCLE datasets. The relevance between AMIGO2 and M2 polarization was then further identified in our local PDAC cohort. Finally, the role of AMIGO2 as cancer promoter and pivotal factor enrolled in M2 polarization was verified through siRNA transfection and M2 macrophages induction. These findings could facilitate diagnosis and treatment of PDAC. In addition, further research was deemed necessary on the deep mechanism between AMIGO2 and M2 polarization of macrophages in PDAC.

## INTRODUCTION

Pancreatic ductal adenocarcinoma (PDAC) is a lethal type of malignant tumor. PDAC is reported to be the seventh major cause of tumor-related death [1]. Around 460,000 patients are diagnosed and more than 400,000 PDAC patients perish due to tumor development every year based on worldwide cancer statistics [1]. In this era of advanced technology, early diagnosis of PDAC still remains difficult and the tumor progression can be rather rapid, with a large possibility of chemotherapy and radiotherapy resistance [2]. However, the development of high-throughput sequencing and bioinformatics provide numerous PDAC-related datasets derived from different stages of tumor samples and normal samples. New biological indicators and novel therapeutic targets based on high-throughput sequencing and bioinformatics are of great need in the current situation of PDAC treatment.

During the tumor immunity, a strong anti-cancer response is activated to identify and kill cells with malignant characteristics and behaviors through the recruitment of different immune cells with immune checkpoint. However, PDAC can be refractory to immune checkpoint blocking therapy [3]. The refraction of PDAC is mainly due to specific tumor microenvironments with significantly immunosuppressive characteristics [4]. The subgroups of macrophages are mainly identified as M1 macrophage (active phenotype) and M2 macrophage (inactive phenotype). A high range of M2 macrophages was identified in PDAC tumor samples and it correlates with unfavorable clinical outcome [5]. However, the stimulator of M2 macrophage and its participation in PDAC need to be identified.

With the development of gene sequencing technology, bioinformatical manipulation was proposed to delineate the characteristic of different types of cancer in multiple biological process [6–8]. However, the efficacy of most gene signatures is not able to meet the expectations of clinical application. In this grim situation, it is necessary to find novel, reliable prognostic biomarkers. Machine learning algorithm is developing rapidly and some robust machine learning method was executed on multiple types of cancers [9–12]. However, to date, combined use of several machine learning and statistical methods in different PDAC sequencing datasets has not been reported.

In our current study, AMIGO2 was identified with significant prognostic and diagnostic value. Our findings were based on the analysis of high throughput datasets from TCGA and GEO database with clinical data, two machine learning methods, and LASSO regression. The potential carcinogenic function of AMIGO2 in PDAC was then predicted *in silico* by pan-cancer analysis and verified in our local PDAC cohorts, two pancreatic cancer cell lines and macrophage cell lines.

## MATERIALS AND METHODS

### High throughput data acquirement

Transcriptional gene matrix of PDAC with related clinical information is downloaded from TCGA data bank (https://www.cancer.gov/about-nci/organization/ccg/research/structural-genomics/tcga) named TCGA-PAAD with 178 cancers and corresponding clinical data), and nine datasets in Gene Expression Omnibus (GEO) database (http://www.ncbi.nlm.nih.gov/geo/), including GSE15471 (36 paracancers 36 cancers), GSE16515 (16 paracancers 36 cancers), GSE28735 (45 paracancers 45 cancers), GSE41368 (6 paracancers 6 cancers), GSE62165 (13 paracancers 118 cancers), GSE62452 (61 paracancers 69 cancers), GSE71989 (8 paracancers 14 cancers), GSE91035 (23 paracancers 27 cancers) and GSE60980 (12 paracancers, 52 cancers). Pan-cancer gene expression datasets (*n* = 7801) were downloaded in UCSC Xena (https://xenabrowser.net/datapages/), named TCGA Pan-Cancer (PANCAN), and pan-normal tissue gene expression datasets (*n* = 7858) were also downloaded in UCSC Xena (https://xenabrowser.net/datapages/), named GTEx. All the gene matrices are downloaded in counts format.

### Batch normalization

To minimize the influence of batch effect among GSE15471 (36 paracancers 36 cancers), GSE16515 (16 paracancers 36 cancers), GSE28735 (45 paracancers 45 cancers), GSE41368 (6 paracancers 6 cancers), GSE62165 (13 paracancers 118 cancers), GSE62452 (61 paracancers 69 cancers), GSE71989 (8 paracancers 14 cancers), and GSE91035 (23 paracancers 27 cancers), SVA package was exerted in R software [13].

### Differential analysis and gene enrichment analysis

Differential expressed genes were acquired through limma package in R software [14], the criteria of differential expressed gene are logFC>1.5 and adj*P* value < 0.05. GO (Gene ontology) analysis, KEGG (Kyoto Encyclopedia of Genes and Genomes) pathway analysis and DO (Disease ontology) analysis were performed using clusterprofile package in R software.

### LASSO regression and SVM-RFE

The differential expressed genes are screened by LASSO regression method with 1000 simulations through “glmnet” package in R software [15]. SVM-RFE (Support Vector Machine-Recursive Feature Elimination) is a sequence backward selection algorithm based on the maximum interval principle of Support Vector Machine [16]. The hub genes combined transcriptional gene matrix of PDAC was filtered through SVM-RFE method. To improve the robust characteristic of the model with minimal error, the screening procedure was exerted in repetition 1000 times.

### Supervised machine learning screening (random forest)

Random forest is a robust clustering algorithm for hub gene identification, and it can be used to calculate the significance of predictive variables distinguished from background noise [17]. In the current study, random forest algorithm was used to identify hub genes in PDAC based on TCGA-PAAD gene expression matrix combining with clinical data through randomForest package in R software [18].

### GSEA (gene set enrichment analysis)

GSEA is based on the idea of using a predefined set of genes, often derived from functional annotations or the results of previous experiments, to enrich genes based on the degree of differential expression between two groups, and the rank of the gene set can be identified. In this research, the PDAC samples in TCGA was divided into two groups, AMIGO2 high expression group and AMIGO2 low expression group based on the median expression of AMIGO2. GSEA was exerted using Java edition of GSEA software (http://www.gsea-msigdb.org/gsea/index.jsp) and GSEA package in R software.

### Cell culture

Pancreatic carcinoma cell lines of Homo sapiens, including SW1990 and MPanc-96 were obtained from Changhai Hospital, Shanghai. Cells were cultured in DMEM with 10% fetal bovine serum (Gibco, USA), 100 μg/mL streptomycin and 100 U/mL penicillin (Invitrogen, USA) at 37°C under 5% CO2 and 1% O2. All the following experiments were independently repeated three times. For inducing M2 polarization of RAW 264.7, 10 ng/ml IL-4 (P00196, Solarbio, China) was added in 6 wells plate 24 hours after RAW 264.7 cells seeded in the plate. Then, the cell was harvested after another 24 hours.

### siRNA and cell transfection

Human AMIGO2 siRNA were obtained from RiboBio (Guangzhou, China). The sequence of the siRNA is 5′-UUAGGAUGCCCUCAGCUAUCACUGC-3′ and 5′-AUUGUUGUAAAGCAGAAGCACUUCC-3. Lipofectamine 3000 Reagent (Invitrogen, USA) was acquired to transfect the cancer cells. 1 μg of siRNA was respectively transfected into SW1990 and MPanc-96, through Lipofectamine 3000 reagent (Thermo Fisher Scientific, USA), with the lead of the manufacturer’s instructions. Then, the cells were cultured for another 24 h and harvested for further analysis. The knockdown efficiency of AMIGO2 in human cancer cells was verified by qRT–PCR.

### Clinical samples collection

A total of 80 frozen primary PDAC samples with paracancerous normal tissue were collected at the Department of Gastrointestinal Surgery, General Hospital of Ningxia Medical University from Jan 2012 to October 2019. The average follow-up time of each patient is 1 year. Consent was acquired from all patients in written format. This study was executed according to Declaration of Helsinki, and the Ethics Committee of General Hospital of Ningxia Medical University. The baseline characteristics of PDAC patients are listed in Table 1.

**Table 1:** Detail clinical data of qRT-PCR data from 80 samples.

	**High risk (*N* = 40)**	**Low risk (*N* = 40)**	***P*-value**	**ALL (*N* = 80)**
Age >65:				
Yes	26 (65%)	28 (70%)	0.57	54 (67.5%)
No	14 (35%)	12 (30%)		26 (32.5%)
Sex:				
Male	20 (50%)	24 (60%)	0.82	44 (55%)
Female	20 (50%)	16 (40%)		36 (45%)
Grade:				
G1	8 (20.0%)	23 (57.5%)	0.02	31 (38.8%)
G2	18 (45.0%)	11 (27.5%)		29 (36.2%)
G3	14 (35.0%)	6 (15%)		20 (25%)
T				
T1	17 (42.5%)	27 (67.5%)	0.04	44 (55%)
T2	10 (25%)	7 (17.5%)		17 (21.3%)
T3	11 (27.5%)	5(12.5%)		16 (20%)
T4	2 (5%)	1(2.5%)		3 (3.7%)
M				
M0	32 (80%)	34 (85%)	0.738	66 (82.5%)
M1	8 (20%)	6 (15%)		14 (17.5%)
N				
N0	27 (67.5%)	29 (72.5%)	0.826	56 (70%)
N1	13 (32.5%)	11 (27.5%)		24 (30%)

### Immunohistochemical staining

Immunohistochemical analysis was conducted as described previously [19]. PDAC tissues were obtained from PDAC patients above and soaked in formalin solution. Then the tissues were embedded in paraffin and cut in pieces after dehydrated. Tissue sections were deparaffinized, rehydrated, and incubated for 30 min in 0.3% hydrogen peroxide in methanol and then for 10 min with 1% goat serum in TBS. After this, the section was incubated with anti-AMIGO2 (821607, Zen-Bio, USA), anti-CSF1R (160600, Zen-Bio) at room temperature overnight. After washing three times in TBS, the sections were soaked with biotinylated mouse anti-rabbit IgG (1:10000; Dingguo Changsheng Company, China) for 1 hours and washed three times in TBS. The final incubation was for 30 min with HRP-avidin D at room temperature. The peroxidase was detected with 0.05% 3,3-diaminobenzidine tetrahydrochloride (DAB). The sections were counterstained with hematoxylin and mounted in neutral gum medium for light microscopy. Positive protein expression was visualized as nuclear localization of granular brown-yellow precipitate.

### RT-PCR and real-time PCR primers

Total RNA was extracted through Trizol reagent (Invitrogen, USA) with the guide of the manufacturer’s instructions and then reverse transcribed into cDNA using a cDNA chain synthesis kit (Vazyme, China). The qPCR experiment was performed using a Light Roche 480 System. The human AMIGO2 RT-PCR forward and reverse primers were 5′-GATACTGCAGCA GGGCAGAA-3′ and 5′-GACGCCACAAAAGGT GTGTC-3′. The mouse Amigo2 RT-PCR forward and reverse primers were 5′-CTTCGCCACAACAAC ATCAC-3′ and 5′-TGGCACTCTTTACCGACTTCA-3′. The mouse Gapdh RT-PCR forward reverse primers were 5′-TGTTCGTCATGGGTGTGAAC-3′ and 5′-ATGGCATGGACTGTGGTCAT -3′. The mouse Mrc1 RT-PCR forward and reverse primers were CTCAACCCAAGGGCTCTTCTAA and AGGTGG CCTCTTGAGGTATGTG. The mouse Csf1r RT-PCR forward and reverse primers were 5′-TGGATGCCT GTGAATGGCTCTG-3′ and 5′-GTGGGTGTCATTCC AAACCTGC-3′.

The mouse IL-10 forward and reverse primers were 5′-ACTGGCATGAGGATCAGCAG-3′, 5′-CTCCTTGA TTTCTGGGCCAT-3′, and mouse TNF-α forward and reverse primers were 5′-TGTAGCCCACGTCG TAGCAAA-3′ and 5′-CTGGCACCACTAGTTGG TTGT-3′, the mouse Gapdh forward and reverse primers were 5′-TGTGATGGGTGTGAACCACG-3′, 5′-CAGTGAGCTTCCCGTTCACC-3′.

### Cell proliferation assay

Cell Counting Kit 8 (Dojindo, Japan) was used to assess cell proliferation. Transfected cells were collected and plated in 96-well culture plates (2 × 10^3^ per well). Cell viability was detected for 5 days following the protocol once a day. The optical density of each well at 450 nm (OD 450 nm) was measured using a microplate reader (Multiskan FC, Thermo Fisher Scientific, USA).

### Western blot assay

RIPA solution (Beyotime, China) with protease inhibitor cocktail (Roche, USA) was used to extract the total protein from cells. BCA Protein Assay Kit (Thermo Fisher Scientific, USA) was used to measure the concentration of the protein above. The protein samples were adjusted to a uniform concentration, separated by 10% SDS-PAGE and transferred to polyvinylidene fluoride membranes (Millipore, USA). Then, 5% nonfat milk was used to block the membranes, and several antibodies were co-incubated with the obtained membrane. The antibodies contain: anti-AMIGO2 (821607, Zen-Bio), anti-CSF1R (160600, Zen-Bio), anti-alpha GAPDH (ab8245, Abcam, UK). Goat anti-Rabbit HRP (Dingguo Changsheng, IH-0011). Finally, an ECL chromogenic machine was used to detect fluorescent signals (Amersham Imager 600, USA). The relative protein expression (AMIGO2 or CSF1R/GAPDH) is calculated by gray level calculation through ImageJ software.

### Statistical analysis and visualization

All statistical analysis of sequencing data and visualization were exerted in R software. In particular, differential analysis of gene sets was calculated using the limma package. Pheatmap package was used to draw heatmap based on sequencing data of pancreatic ductal adenocarcinoma. Clusterprofile package was used to exert GO and KEGG analysis. LASSO regression was exerted using the Glmnet package. Pearson correlation coefficient was calculated based on the expression data of Pancreatic ductal adenocarcinoma, pancancers, and normal samples in TCGA, GEO and GTEx database. Wilcoxon rank sum test and *T* test was exerted in our local patient cohort. The result of qPCR was visualized and regression coefficient was calculated in GraphPad Prism 7.0.

### Online tool

Kaplan-Meier survival analysis and differential analysis of AMIGO2 was exerted through two online database, GEPIA (http://gepia.cancer-pku.cn/) and KM plotter (https://kmplot.com/analysis/), based on Pancreatic ductal adenocarcinoma data in TCGA and GEO database.

## RESULTS

### Identification of differential expressed genes and pivotal pathways

**Figure 1:**
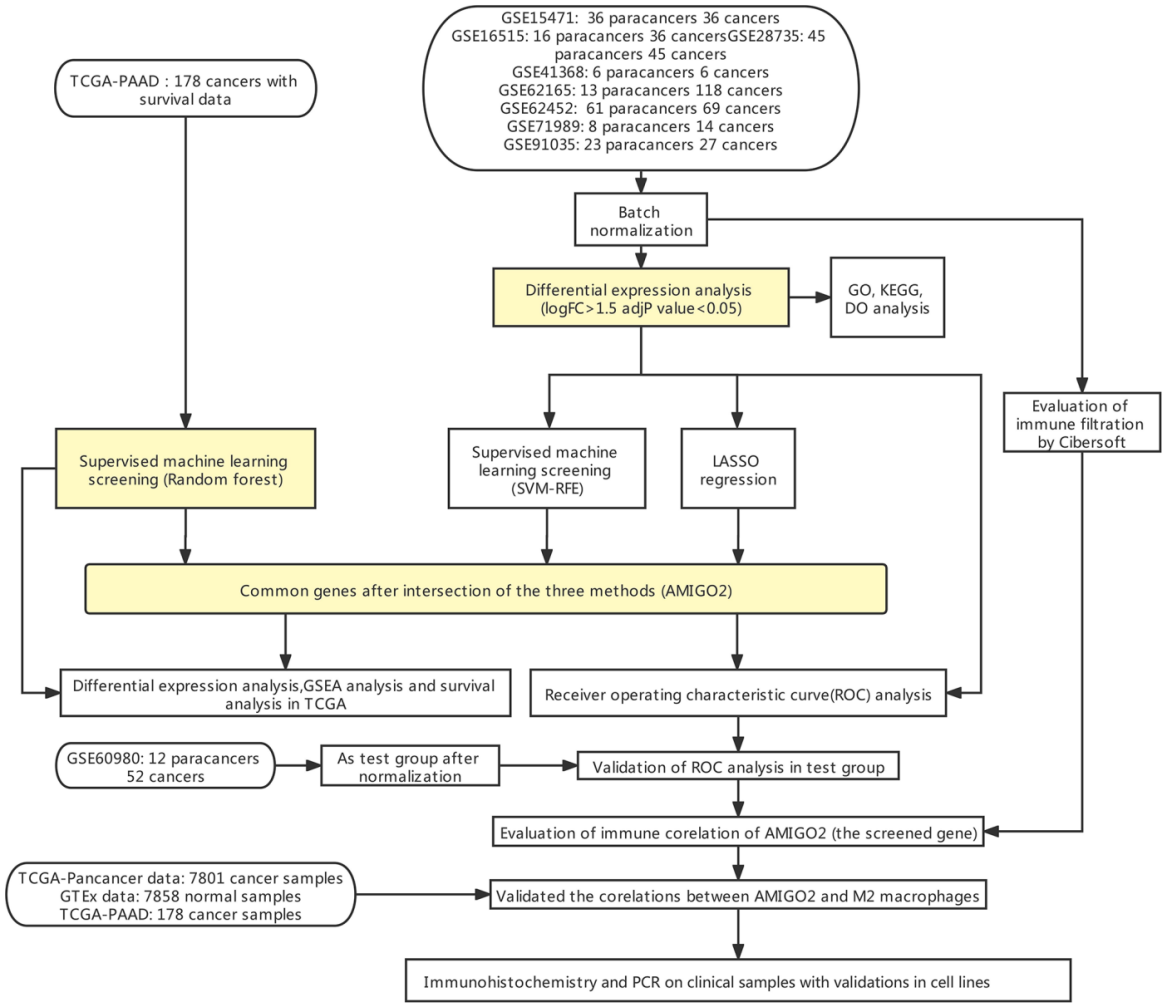
Flow chart of the current study.

Figure 1 indicates the procedure of our study. Firstly, 8 datasets were enrolled in this study, including GSE15471 (36 paracancers 36 cancers), GSE16515 (16 paracancers 36 cancers), GSE28735 (45 paracancers 45 cancers), GSE41368 (6 paracancers 6 cancers), GSE62165 (13 paracancers 118 cancers), GSE62452 (61 paracancers 69 cancers), GSE71989 (8 paracancers 14 cancers), GSE91035 (23 paracancers 27 cancers) and GSE60980 (12 paracancers, 52 cancers).

**Figure 2:**
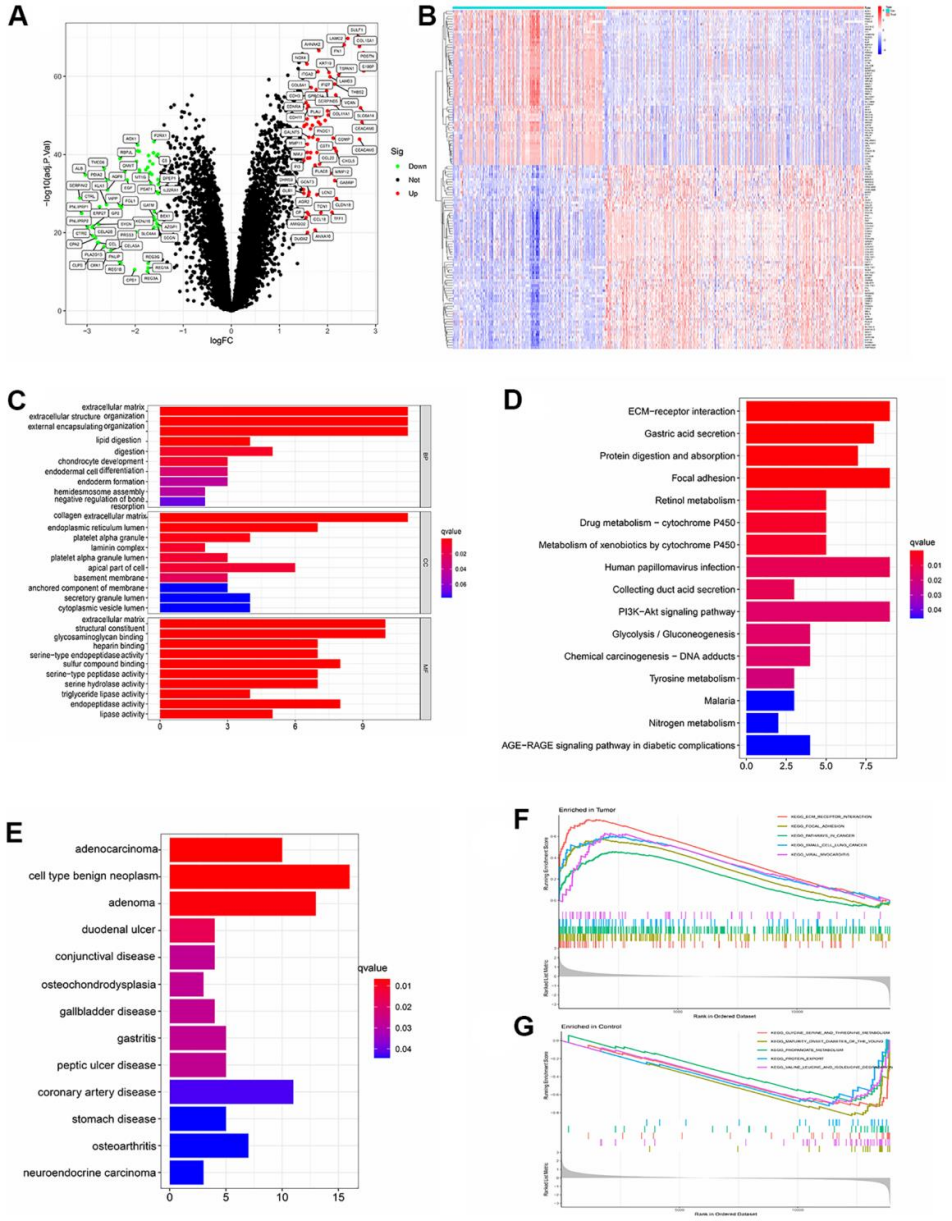
(**A**) Volcano plot showed significant differential expressed genes. (logFC>1.5 adj. *P* value < 0.05). (**B**) Heatmap showed the expression distribution of differential expressed genes in each sample. (**C**) Gene ontology (GO) analysis of the differential expressed genes. (**D**) KEGG analysis of the differential expressed genes. (**E**) Disease ontology (DO) analysis of the differential expressed genes. (**F**) GSEA result in tumor samples. (**G**) GSEA result in paired paracancerous samples.

After batch normalization and differential gene analysis, 60 down-expressed genes and 71 up-expressed genes was identified (Supplementary Table 1 and Figure 2A, 2B). In GO analysis, some cancer-related terms, including “extracellular matrix organization”, “lipid digestion”, “serine-type endopeptidase activity” were identified as enriched terms (Figure 2C). In KEGG (Kyoto Encyclopedia of Genes and Genomes) analysis, the cancer-associated pathway, like “ECM-receptor interaction”, “Focal adhesion”, “PI3K-Akt signaling pathway”, “Glycolysis/Gluconeogenesis”, “Chemical carcinogenesis - DNA adducts” was enriched (Figure 2D). In DO analysis, “adenocarcinoma”, “cell type benign neoplasm”, “neuroendocrine carcinoma” was identified (Figure 2E). “KEGG_ECM_RECEPTOR_ INTERACTION”, “KEGG_FOCAL_ADHESION”, “KEGG_PATHWAYS_IN_CANCER” were identified in tumor samples of PDAC patients. “KEGG_GLYCINE_SERINE_AND_THREONINE_METABOLISM”, “KEGG_PROTEIN_EXPORT” and some other pathways were identified using GSEA in normal samples of PDAC patients (Figure 2F, 2G and Supplementary Table 2).

### Screening pivotal genes in PDAC through multiple machine learning methods in two PDAC cohorts

**Figure 3:**
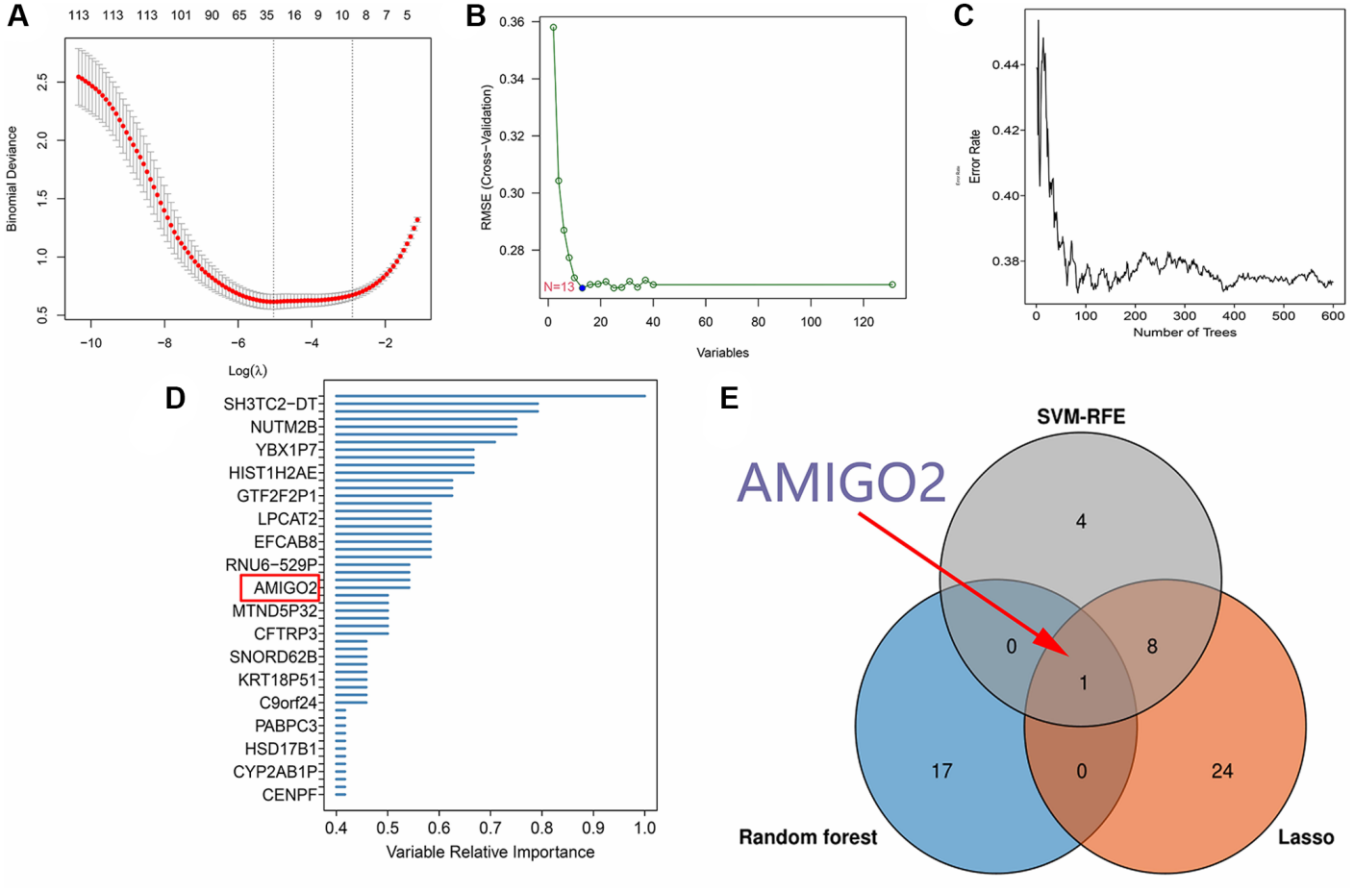
(**A**) 33 genes are screened through LASSO regression in combined GEO datasets. (**B**) 13 genes are screened through SVM-RFE in combined GEO datasets. (**C**, **D**) 18 genes are screened through Random forest in TCGA-PAAD datasets. (**E**) Venn plot shows the intersected genes.

After identification of significant pathway in differential expressed genes, further screening procedure based on LASSO regression and machine learning method was exerted. 33 genes are screened through LASSO regression in combined GEO datasets (Figure 3A and Supplementary Table 3). 13 genes are screened through SVM-RFE in combined GEO datasets (Figure 3B and Supplementary Table 4). 18 genes are screened through Random forest in TCGA-PAAD datasets (Figure 3C, 3D). Combining the findings above, AMIGO2 was identified as the common intersected genes among three procedures (Figure 3E).

### The diagnostic value, prognostic value and significant enriched pathway of AMIGO2 in PDAC

**Figure 4:**
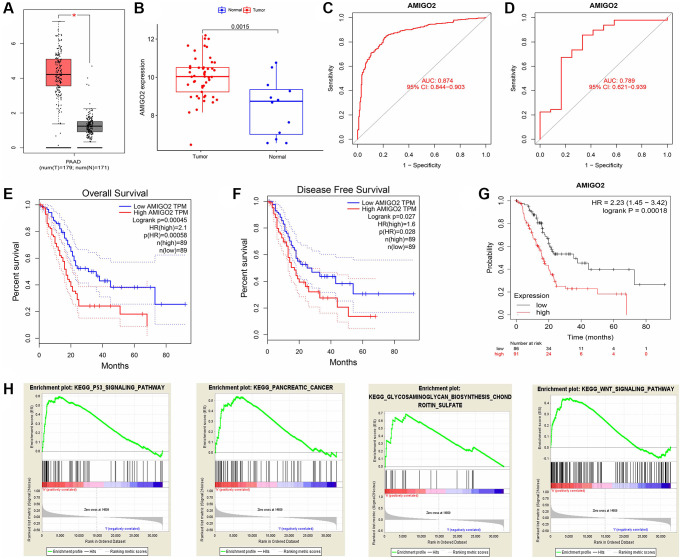
(**A**) The expression of AMIGO2 between normal samples and tumor samples of PDAC patients in TCGA datasets. (**B**) The expression of AMIGO2 between normal samples and tumor samples of PDAC patients in GSE60980 (Validation group). (**C**) The ROC curve of AMIGO2 in the combined GEO datasets group above. (**D**) The ROC curve of AMIGO2 in GSE60980 (Validation group). (**E**) Overall survival of PDAC patients with different expression of AMIGO2 in GEPIA. (**F**) Disease free survival of PDAC patients with different expression of AMIGO2 in GEPIA. (**G**) Overall survival of PDAC patients with different expression of AMIGO2 in KM plotter. (**H**) The GSEA results comparing AMIGO2-high group to AMIGO2-low group.

Then, the significant differential expression of AMIGO2 in PDAC was validated in TCGA datasets (Figure 4A) and GSE60980 (Figure 4B). The ROC curve of AMIGO2 in former combined group is 0.824 (Figure 4C), and the ROC curve of AMIGO2 in GSE60980 is 0.724 (Figure 4D). The prognostic value of AMIGO2 in PDAC was validated in GEPIA and KM plotter (Figure 4E–4G) Four representative significant enriched pathway (*P* < 0.05) related to AMIGO2 in PDAC was identified, including “KEGG_P53_SIGNALING_PATHWAY”, “KEGG_ PANCREATIC_CANCER”, “KEGG_GLYCOSAMIN OGLYCAN_BIOSYNTHESIS_CHONDROITIN_SULFATE”, “KEGG_WNT_SIGNALING_PATHWAY” (Figure 4H). The details of these GSEA results are shown in Supplementary Table 5.

### The immune microenvironment of PDAC and the correlation with AMIGO2

**Figure 5:**
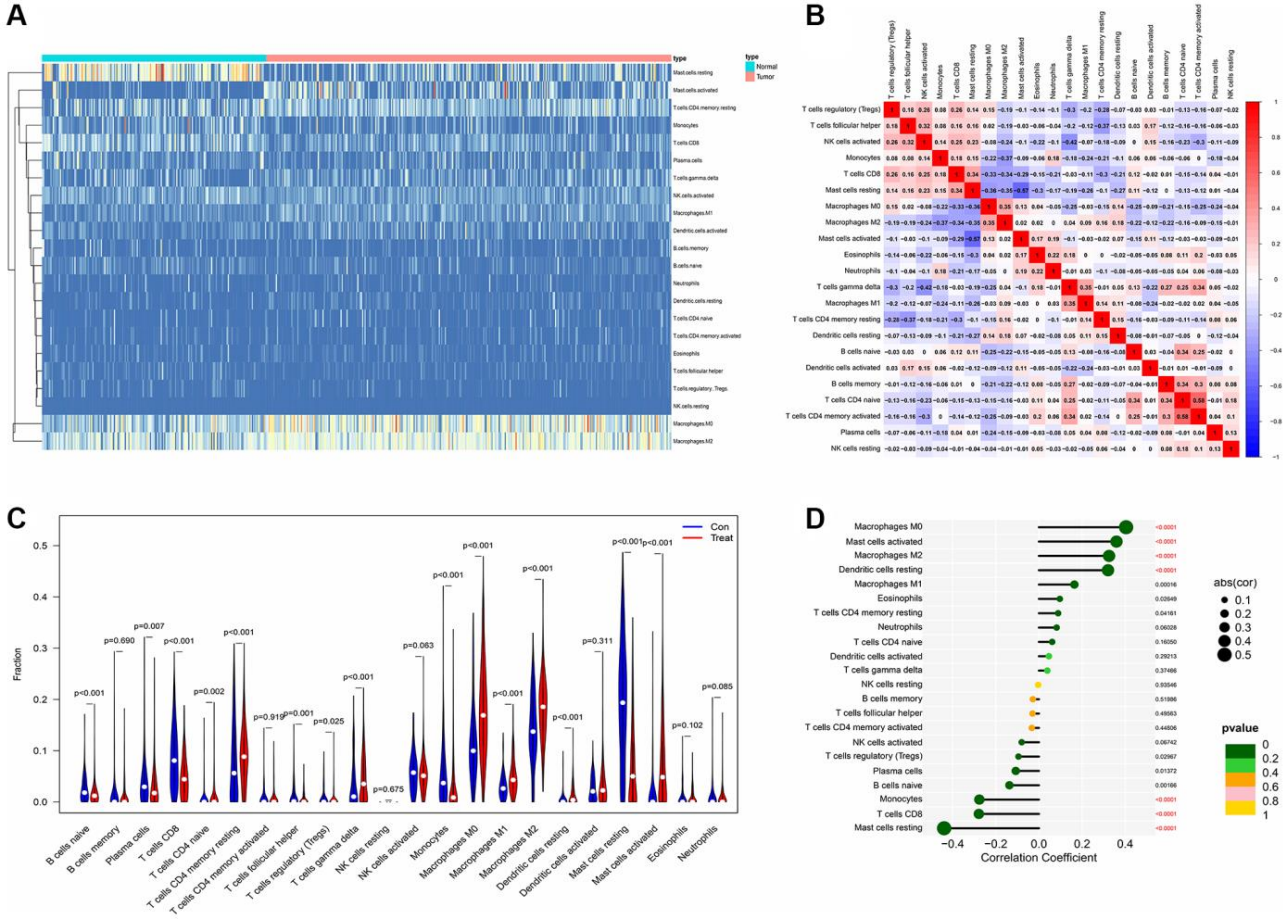
(**A**) The distribution of each immune subtype between normal samples and tumor samples in PDAC. (**B**) The correlation among each immune subtype in PDAC tumor samples in former combined GEO datasets. (**C**) The comparation of immune cell types between normal samples and tumor samples in different immune subtypes of PDAC in former combined GEO datasets. (**D**) The correlation coefficient between AMIGO2 and main immune cell types in GEO datasets.

Tumor microenvironment analysis in former combined GEO datasets was exerted through “cibersoft” algorithm. Resting mast cells were enriched in paracancerous normal samples and rarely existed in cancer samples. More importantly, M1 macrophages were hardly enriched in both paracancerous normal samples and tumor samples of PDAC patients. On the contrary, M0 and M2 macrophages are existed in paracancerous normal samples and tumor samples of PDAC patients in ubiquity (Figure 5A). In addition, M0 and M2 macrophages are significantly negatively correlated to CD8 T cells, which is a mainly effector of anti-tumor immunity (Figure 5B). Moreover, M2 and M0 macrophage significantly increased in tumor samples of PDAC patients, compared to normal tissue of PDAC patients. Although M1 macrophage is significantly enriched in PDAC tumor samples, comparing with the range of change in M0 and M2 macrophages, the range of M1 macrophages between normal and tumor tissue is compromised (Figure 5C). Combining with the expression of AMIGO2, the increase of AMIGO2 in tumor tissues is significantly correlated to M0 and M2 macrophages (*P* < 0.0001, Figure 5D).

### Validation of the correlation between M2 macrophages and AMIGO2 *in silico*

**Figure 6:**
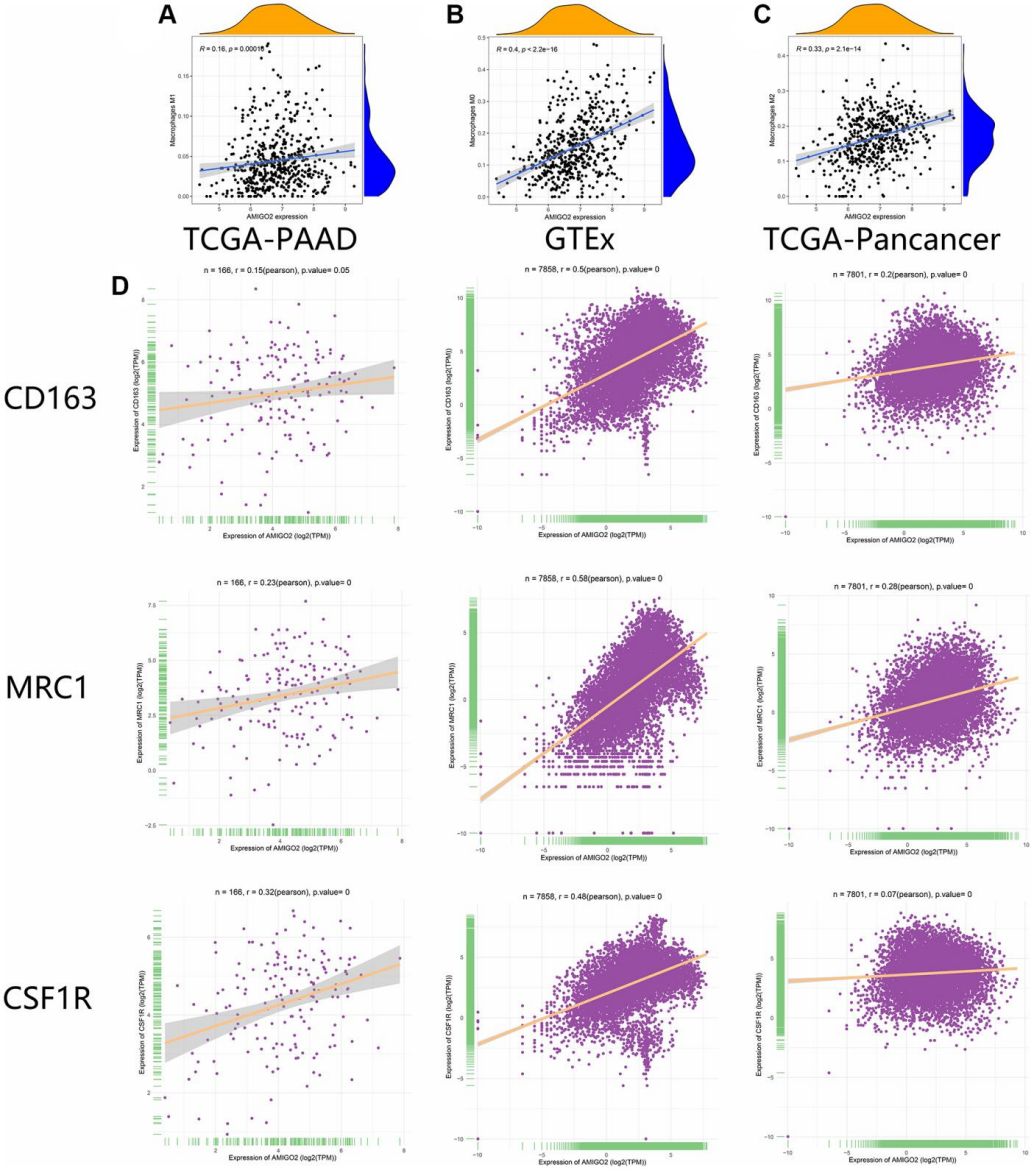
(**A**) The correlation between the expression of AMIGO2 and M1 macrophages infiltration in former combined GEO datasets. (**B**) The correlation between the expression of AMIGO2 and M0 macrophages infiltration in former combined GEO datasets. (**C**) The correlation between the expression of AMIGO2 and M2 macrophages infiltration in former combined GEO datasets. (**D**) The correlations between 3 M2 macrophage biomarkers and AMIGO2 expression in TCGA-PAAD, TCGA-Pancancer and GTEx datasets.

As shown in Figure 6A–6D, the correlations between AMIGO2 expression and M0 and M2 macrophages are significantly higher than the correlation between AMIGO2 expression and M1 macrophage. Then three biomarkers of M2 macrophage (CD163, MRC1, CSF1R) was chosen to identify the correlation between AMIGO2 expression and M2 macrophage in large scale of high-throughput data. AMIGO2 expression is significantly correlated to the polarization of M2 macrophage in pancancer datasets, PDAC datasets and normal tissue datasets.

### Validation of the correlation between M2 macrophages and AMIGO2 in our local cohort

**Figure 7:**
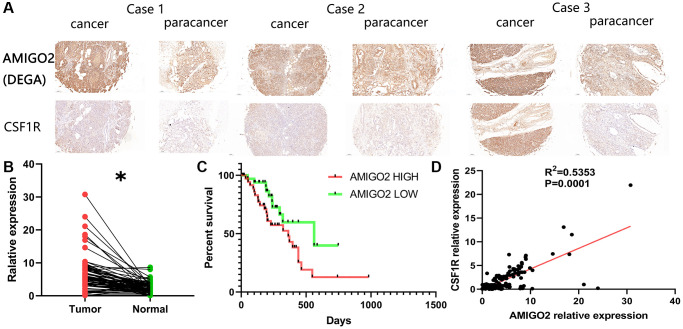
(**A**) Representative immunohistochemical imagines of AMIGO2 and CSF1R in cancer and paracancerous tissue; ^*^p < 0.05. (**B**) The mRNA expression of AMIGO2 in caner and paracancerous tissue. (**C**) Survival analysis of AMIGO2. (**D**) The correlation between the mRNA expression of CSF1R and AMIGO2 in cancer and paracancerous tissue.

Then, our local cohort with 80 PDAC patients was enrolled in the current study. Specifically, the 80 clinical samples were divided into high-risk group (AMIGO2 high) and low risk group (AMIGO2 low) according to the median expression level of AMIGO2 (Table 1). Immunohistochemical imagines of cancer and paracancer shows AMIGO2 and CSF1R are up-regulated on PDAC tissue compare to paracancer tissue (Figure 7A). AMIGO2 is significantly up-regulated on PDAC tissue in our cohort (Figure 7B) according to our qPCR results. Up-regulated AMIGO2 also indicates poor clinical outcomes in our current cohort (Figure 7C). Interestingly, a robust correlation between the expression of CSF1R and AMIGO2 in cancer and paracancer tissue are also identified (Figure 7D).

### Knockdown of AMIGO2 restrain the proliferation of pancreatic ductal adenocarcinoma cells and AMIGO2 was upregulated in M2 macrophages

**Figure 8:**
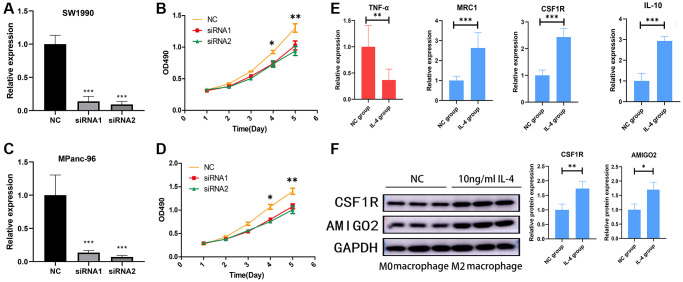
(**A**) The mRNA expression of AMIGO2 in normal control SW1990 cell line and two AMIGO2 siRNA transfected SW1990 cell line; ^*^p < 0.05, ^**^p < 0.01, ^***^p < 0.001. (**B**) Relative proliferation tendency in normal control SW1990 cell line and two AMIGO2 siRNA transfected SW1990 cell line; ^*^p < 0.05, ^**^p < 0.01, ^***^p < 0.001. (**C**) The mRNA expression of AMIGO2 in normal control MPanc-96 cell line and two AMIGO2 siRNA transfected MPanc-96 cell line; ^*^p < 0.05, ^**^p < 0.01, ^***^p < 0.001. (**D**) Relative proliferation tendency in normal control MPanc-96 cell line and two AMIGO2 siRNA transfected MPanc-96 cell line; ^*^p < 0.05, ^**^p < 0.01, ^***^p < 0.001. (**E**) The mRNA expression of IL-10, MRC1, CSF1R and TNF-α in untreated and IL-4 treated RAW264.7 cell, ^*^p < 0.05, ^**^p < 0.01, ^***^p < 0.001. (**F**) The protein level of AMIGO2 and CSF1R in untreated and IL-4 treated RAW264.7 cell, ^*^p < 0.05, ^**^p < 0.01, ^***^p < 0.001.

Then, AMIGO2 siRNA was successfully transfected in two Pancreatic ductal adenocarcinoma cell lines, SW1990 and MPanc-96 (Figure 8A, 8C). CCK8 assay shows knock out of AMIGO2 restrains the proliferation of Pancreatic ductal adenocarcinoma cell (Figure 8B, 8D). After inducing M2 polarization of macrophages by adding IL-4 in RAW264.7 cell, the mRNA expression of TNF-α (biomarker of M1 macrophage) in IL-4 treated group is significantly decreased compared to untreated group (NC group, *P* < 0.01), and the mRNA expression of M2 macrophage biomarkers, including IL-10, CSF1R and MRC1 in IL-4 treated group is significantly increased compared to untreated group (NC group, Figure 8E, *P* < 0.01). After confirmation of IL-4 induced M2 macrophage, the protein level of M2 polarization biomarker, CSF1R, and AMIGO2 was detected in M2 macrophages (IL-4 treated group) and M0 macrophages (NC group). The expression of CSF1R and AMIGO2 was significantly increased in M2 macrophages (Figure 8F, *p* < 0.05).

## DISCUSSION

PDAC is a fatal carcinoma with loose texture of cancer tissue and infiltration of a large number of immune cells. During PDAC related immune response, a robust anti-cancer mechanism is activated to identify and eliminate those cells which have malignant tendency. Based on the anti-tumor immunity, immune checkpoint blocking therapy, including anti PD-1, anti PD-L1 and anti CTLA-4 shows great efficacy on a large amount of solid tumors. However, recently published clinical trials reported that PDAC can be refractory to immune checkpoint blocking therapy [20–22]. The composition of immune cells in PDAC tumor microenvironment is diverse, including CD4+ T cells, CD8+ T cells, cancer associated fibroblast cells, and different kinds of macrophages. These kinds of cells take part in the refractory phenotypes of PDAC [23]. The subgroups of macrophages are mainly identified as M1 macrophage and M2 macrophage. M1 macrophage exhibits an inflammation-related phenotype which is rare in PDAC tumor microenvironment. The biomarkers of M1 macrophages include multiple pro-inflammatory cytokines, including TNF-αand IL-1 β. On the other hand, M2 macrophages play an important role as an immune suppressor, tumor-associated angiogenesis stimulator [24], epithelial-mesenchymal transition (EMT) promoter, and an accelerator of malignant phenotype in PDAC cell lines. The biomarker of M2 macrophages includes multiple anti-inflammatory cytokines, including IL-10, CSF1R, and MRC1 [6, 25, 26]. In addition, a high range of M2 macrophages was identified in PDAC tumor samples and it correlates with unfavorable clinical outcome [5].

AMIGO2 (Adhesion Molecule with Ig Like Domain 2), also called DEGA (Differentially Expressed in Gastric Adenocarcinoma) was found to play a potential role in carcinogenesis in 2004 [27]. Since then, several studies discussed the specific cancer-related biological function of the AMIGO2. Down-regulation of the AMIGO2 limits proliferation, migration, and invasion in gastric cancer cell lines [27]. Up-regulation of AMIGO2 is related to terminal stage of colon cancer patients [28]. AMIGO2 also acts as a key promoter of malignant phenotype in breast cancer [29]. After knockout of AMIGO2 in ovarian cancer cells, the capacity of cell migration, invasion *in vitro* and metastasis *in vivo* is significantly compromised [30].

In our current study, we combined 8 datasets with PADC high-throughput sequencing data in GEO datasets, and differential expressed genes with significant pathway and enriched related diseases are identified through bioinformatical analysis. LASSO regression analysis and SVM-RFE machine learning method was then exerted. After combination of Random Forest machine learning method based on PDAC data from TCGA database, AMIGO2 gene was identified as a pivotal gene in PDAC carcinogenesis. The diagnostic and prognostic values of the AMIGO2 were identified in two independent PDAC cohorts. The correlation of M2 polarization of macrophage and AMIGO2 was identified and validated in both clinical samples and cell lines. Previous studis have reported that AMIGO2 could active PDK1/Akt pathway [31, 32], and it is well-known that activation of Akt pathway is a pivotal factor for M2 polarization in macrophage. We speculate that the upregulation of AMIGO2 actives M2 polarization of macrophages through Akt pathway. However, further experiments are deemed necessary to verify this hypothesis.

Previous studies have reported on the few significant gene cluster and gene signatures with diagnostic and prognostic value through machine learning methods in Pancreatic ductal adenocarcinoma [33, 34]. However, to the best of our knowledge, the analysis based on the combination of all main pancreatic ductal adenocarcinoma datasets in GEO and TCGA has not been exerted yet. Additionally, through the combination use of several machine learning methods on multiple pancreatic ductal adenocarcinoma datasets, AMIGO2 is identified as a significant diagnostic and prognostic biomarker in PDAC. The competence of macrophage M2 polarization and the role as cancer promoter was identified through Pearson relevant analysis based on the pancancer tissues and normal tissues (more than 10000 samples), and verified on biological experiment, including M2 polarization inducing on macrophage and proliferation analysis on cancer cell. These highlights add academic value of our study and could guide the fellow researches based on the combination of bioinformatical analysis and bioinformatics-guided biological experiment.

## CONCLUSION

Our findings identified the potential pivotal roles of AMIGO2 in PDAC carcinogenesis and M2 polarization with significant diagnostic and prognostic value. These findings could facilitate precise treatment of PDAC at molecular level and instruct more tumor immunity-related studies focusing on the biological function between AMIGO2 and M2 macrophages in PDAC. However, the deep mechanism of AMIGO2 in pancreatic ductal adenocarcinoma cells and M2 polarization of macrophage needed to be further clarified and should be the focus of future research.

## Supplementary Materials

Supplementary Table 1

Supplementary Table 2

Supplementary Tables 3 and 4

Supplementary Table 5
