# Supplementary material for: AMIGO2 is a pivotal therapeutic target related to M2 polarization of macrophages in pancreatic ductal adenocarcinoma
Source: Aging (Albany NY). 2024 Jan 5;16(2):1111–27. doi: 10.18632/aging.205380 (PMC10866418; doi:10.18632/aging.205380)
Supplement: Supplementary Table 1 [file aging-16-205380-s001.docx]

Supplementary Table 1. Different expressed genes between pancreatic cancer and normal tissue.

| id | logFC | AveExpr | t | P.Value | adj.P.Val | B |
| --- | --- | --- | --- | --- | --- | --- |
| SULF1 | 2.639401 | 8.869985 | 21.4345 | 4.51E-75 | 6.27E-71 | 160.2577 |
| LAMC2 | 2.422727 | 6.921108 | 21.27926 | 2.84E-74 | 1.98E-70 | 158.4301 |
| FN1 | 2.348146 | 9.743173 | 20.92746 | 1.83E-72 | 8.48E-69 | 154.2938 |
| COL10A1 | 2.665073 | 7.401068 | 20.82487 | 6.15E-72 | 2.14E-68 | 153.0891 |
| AHNAK2 | 1.821907 | 5.911497 | 20.59758 | 9.01E-71 | 2.51E-67 | 150.4229 |
| POSTN | 2.749066 | 8.584932 | 20.47304 | 3.91E-70 | 9.07E-67 | 148.9636 |
| NOX4 | 1.5735 | 5.196317 | 19.87362 | 4.52E-67 | 6.99E-64 | 141.9592 |
| S100P | 2.754084 | 8.095076 | 19.51135 | 3.15E-65 | 3.99E-62 | 137.7431 |
| ITGA2 | 1.801067 | 7.484611 | 19.46368 | 5.50E-65 | 6.38E-62 | 137.1895 |
| KRT19 | 2.031822 | 8.918634 | 19.41703 | 9.49E-65 | 1.02E-61 | 136.6479 |
| TSPAN1 | 2.233092 | 7.845882 | 19.28817 | 4.28E-64 | 4.25E-61 | 135.1531 |
| LAMB3 | 2.053035 | 7.087362 | 19.184 | 1.44E-63 | 1.25E-60 | 133.9463 |
| IFI27 | 1.898134 | 8.212636 | 18.90553 | 3.68E-62 | 2.70E-59 | 130.7269 |
| THBS2 | 2.145852 | 8.997361 | 18.79169 | 1.38E-61 | 9.61E-59 | 129.4138 |
| COL8A1 | 1.661878 | 5.779829 | 18.60781 | 1.16E-60 | 7.37E-58 | 127.2967 |
| GPRC5A | 1.76393 | 6.397794 | 18.49547 | 4.27E-60 | 2.59E-57 | 126.0056 |
| SERPINB5 | 1.980277 | 5.082833 | 18.43269 | 8.83E-60 | 5.12E-57 | 125.2849 |
| VCAN | 2.172176 | 9.38841 | 18.20206 | 1.26E-58 | 6.06E-56 | 122.6427 |
| CDH3 | 1.614192 | 6.318798 | 18.17682 | 1.69E-58 | 7.34E-56 | 122.3541 |
| EDNRA | 1.553912 | 6.677861 | 17.78078 | 1.59E-56 | 5.15E-54 | 117.8389 |
| COL11A1 | 2.162777 | 6.467567 | 17.65667 | 6.58E-56 | 1.99E-53 | 116.4296 |
| PLAU | 1.595797 | 7.284451 | 17.61611 | 1.05E-55 | 2.95E-53 | 115.9696 |
| CDH11 | 1.607893 | 7.197413 | 17.49679 | 4.08E-55 | 1.05E-52 | 114.6183 |
| SLC6A14 | 2.688336 | 5.519568 | 17.48365 | 4.74E-55 | 1.16E-52 | 114.4697 |
| NQO1 | 1.580248 | 7.179056 | 17.44599 | 7.27E-55 | 1.74E-52 | 114.0438 |
| TMPRSS4 | 2.002678 | 6.163601 | 17.31922 | 3.07E-54 | 6.90E-52 | 112.6123 |
| FAP | 2.013968 | 7.979069 | 17.09854 | 3.75E-53 | 7.79E-51 | 110.128 |
| COL5A2 | 1.839905 | 8.037896 | 17.05855 | 5.90E-53 | 1.17E-50 | 109.6789 |
| SDR16C5 | 1.757005 | 5.112871 | 16.97706 | 1.48E-52 | 2.77E-50 | 108.7647 |
| ANLN | 1.57613 | 5.057157 | 16.88008 | 4.42E-52 | 7.88E-50 | 107.6786 |
| COL1A1 | 1.952082 | 9.750145 | 16.79644 | 1.13E-51 | 1.90E-49 | 106.7435 |
| SLPI | 1.811484 | 8.613849 | 16.70596 | 3.13E-51 | 5.16E-49 | 105.7338 |
| CEACAM6 | 2.716329 | 8.482191 | 16.70435 | 3.19E-51 | 5.16E-49 | 105.7158 |
| TRIM29 | 1.567853 | 5.628244 | 16.59338 | 1.11E-50 | 1.73E-48 | 104.4799 |
| COL3A1 | 1.851592 | 10.86254 | 16.58913 | 1.16E-50 | 1.80E-48 | 104.4326 |
| SFN | 1.685858 | 7.132349 | 16.52567 | 2.36E-50 | 3.35E-48 | 103.7272 |
| COL5A1 | 1.521385 | 7.77522 | 16.51277 | 2.73E-50 | 3.84E-48 | 103.5839 |
| AEBP1 | 1.512115 | 9.123088 | 16.46052 | 4.90E-50 | 6.55E-48 | 103.0039 |
| COL12A1 | 1.64711 | 7.291618 | 16.32895 | 2.13E-49 | 2.57E-47 | 101.5463 |
| GALNT5 | 1.615623 | 5.44141 | 15.85664 | 3.99E-47 | 3.88E-45 | 96.34915 |
| FNDC1 | 1.694265 | 7.058595 | 15.85502 | 4.06E-47 | 3.92E-45 | 96.33138 |
| COMP | 2.117911 | 7.424204 | 15.81052 | 6.63E-47 | 6.15E-45 | 95.84473 |
| CEACAM5 | 2.666865 | 6.921888 | 15.74335 | 1.39E-46 | 1.21E-44 | 95.11102 |
| GREM1 | 1.800297 | 7.815187 | 15.72817 | 1.64E-46 | 1.43E-44 | 94.94539 |
| DKK1 | 1.794351 | 5.474609 | 15.71793 | 1.84E-46 | 1.58E-44 | 94.83378 |
| P2RX1 | -1.60811 | 5.661853 | -15.6054 | 6.31E-46 | 5.13E-44 | 93.60793 |
| MMP11 | 1.581489 | 6.99186 | 15.45992 | 3.10E-45 | 2.32E-43 | 92.02892 |
| AOX1 | -1.94684 | 6.806436 | -15.4177 | 4.91E-45 | 3.57E-43 | 91.57218 |
| CST1 | 2.153171 | 6.293647 | 15.22043 | 4.20E-44 | 2.70E-42 | 89.44223 |
| SLC39A5 | -1.94909 | 5.892759 | -15.0828 | 1.86E-43 | 1.17E-41 | 87.96342 |
| CXCL5 | 2.182506 | 6.480725 | 15.06103 | 2.36E-43 | 1.46E-41 | 87.72998 |
| PAIP2B | -1.91357 | 5.888888 | -15.0502 | 2.65E-43 | 1.64E-41 | 87.61425 |
| NMU | 1.581849 | 5.034363 | 15.00002 | 4.55E-43 | 2.72E-41 | 87.07655 |
| COL17A1 | 1.78234 | 5.978747 | 14.99207 | 4.96E-43 | 2.95E-41 | 86.9915 |
| TNFAIP6 | 1.650632 | 6.482092 | 14.982 | 5.52E-43 | 3.22E-41 | 86.88386 |
| ACADL | -1.6183 | 5.010082 | -14.9693 | 6.34E-43 | 3.66E-41 | 86.74752 |
| BACE1 | -1.52565 | 7.094528 | -14.8842 | 1.58E-42 | 8.84E-41 | 85.83895 |
| SLC43A1 | -1.72079 | 7.116357 | -14.8172 | 3.25E-42 | 1.76E-40 | 85.12611 |
| C5 | -1.55601 | 5.814814 | -14.809 | 3.55E-42 | 1.91E-40 | 85.03792 |
| TMC5 | 1.631725 | 6.695797 | 14.67542 | 1.48E-41 | 7.47E-40 | 83.61986 |
| MATN3 | 1.588307 | 5.364271 | 14.6656 | 1.65E-41 | 8.14E-40 | 83.51582 |
| RBPJL | -2.31613 | 5.6953 | -14.6329 | 2.33E-41 | 1.13E-39 | 83.16945 |
| PI3 | 1.508288 | 5.513259 | 14.52544 | 7.32E-41 | 3.28E-39 | 82.03402 |
| PDK4 | -1.69468 | 8.302959 | -14.4766 | 1.23E-40 | 5.28E-39 | 81.51939 |
| ANPEP | -1.97128 | 7.265495 | -14.4692 | 1.33E-40 | 5.68E-39 | 81.44167 |
| F11 | -1.63372 | 4.913093 | -14.3204 | 6.44E-40 | 2.53E-38 | 79.87776 |
| CCL20 | 1.835887 | 6.048339 | 14.30078 | 7.92E-40 | 3.08E-38 | 79.67245 |
| PLAC8 | 1.741367 | 6.802568 | 14.23861 | 1.52E-39 | 5.75E-38 | 79.022 |
| TRHDE | -1.737 | 5.04955 | -14.1135 | 5.68E-39 | 2.00E-37 | 77.7174 |
| TMED6 | -2.64008 | 6.563141 | -14.0414 | 1.21E-38 | 4.07E-37 | 76.96766 |
| GPHA2 | -1.77768 | 5.733588 | -13.9668 | 2.64E-38 | 8.55E-37 | 76.19368 |
| PM20D1 | -1.91386 | 4.983293 | -13.9582 | 2.88E-38 | 9.31E-37 | 76.10505 |
| NR5A2 | -1.90908 | 6.432419 | -13.8961 | 5.52E-38 | 1.71E-36 | 75.46225 |
| GNMT | -2.21834 | 5.497516 | -13.8381 | 1.01E-37 | 3.01E-36 | 74.86441 |
| GUCA1C | -1.69168 | 4.053849 | -13.7864 | 1.72E-37 | 4.97E-36 | 74.33161 |
| MMP12 | 2.061618 | 5.750619 | 13.74895 | 2.54E-37 | 7.20E-36 | 73.94637 |
| NRG4 | -1.65247 | 4.759964 | -13.6961 | 4.39E-37 | 1.23E-35 | 73.40407 |
| ALB | -3.0302 | 6.268358 | -13.6658 | 6.01E-37 | 1.65E-35 | 73.09285 |
| HOMER2 | -1.52375 | 5.893856 | -13.3882 | 1.04E-35 | 2.47E-34 | 70.26372 |
| PDIA2 | -2.58078 | 6.697322 | -13.3688 | 1.27E-35 | 2.99E-34 | 70.06723 |
| EGF | -2.16434 | 5.449812 | -13.3572 | 1.43E-35 | 3.35E-34 | 69.94968 |
| GABRP | 2.151042 | 6.261694 | 13.32295 | 2.03E-35 | 4.70E-34 | 69.60294 |
| MT1G | -1.67392 | 9.156933 | -13.1796 | 8.72E-35 | 1.90E-33 | 68.15768 |
| DPEP1 | -1.51628 | 6.522144 | -13.0959 | 2.04E-34 | 4.27E-33 | 67.3169 |
| DHRS9 | 1.500408 | 5.784764 | 12.77612 | 5.05E-33 | 9.41E-32 | 64.13312 |
| AQP8 | -2.57449 | 6.359292 | -12.7572 | 6.10E-33 | 1.13E-31 | 63.94597 |
| PSAT1 | -1.53931 | 6.152591 | -12.6172 | 2.45E-32 | 4.27E-31 | 62.56712 |
| GCNT3 | 1.618923 | 7.462604 | 12.61662 | 2.47E-32 | 4.29E-31 | 62.56123 |
| OLR1 | 1.509547 | 5.792312 | 12.53973 | 5.28E-32 | 8.90E-31 | 61.80751 |
| IL22RA1 | -1.51832 | 6.071688 | -12.534 | 5.58E-32 | 9.40E-31 | 61.75178 |
| LCN2 | 1.779035 | 8.840377 | 12.45347 | 1.24E-31 | 2.00E-30 | 60.96487 |
| AGR2 | 1.606246 | 7.580052 | 12.42699 | 1.60E-31 | 2.56E-30 | 60.70688 |
| SERPINI2 | -3.15278 | 6.694496 | -12.2482 | 9.21E-31 | 1.37E-29 | 58.97362 |
| CLDN18 | 2.184998 | 6.178351 | 12.21313 | 1.30E-30 | 1.92E-29 | 58.63491 |
| KLK1 | -2.59631 | 8.041686 | -11.8274 | 5.35E-29 | 6.95E-28 | 54.95105 |
| IAPP | -2.31836 | 6.114547 | -11.7784 | 8.53E-29 | 1.09E-27 | 54.48808 |
| ERP27 | -2.73796 | 7.997215 | -11.6908 | 1.96E-28 | 2.42E-27 | 53.66394 |
| FGL1 | -2.28163 | 6.003714 | -11.6667 | 2.46E-28 | 3.02E-27 | 53.4376 |
| CTRL | -3.00267 | 7.753392 | -11.5866 | 5.26E-28 | 6.25E-27 | 52.68755 |
| BEX1 | -1.60351 | 7.305972 | -11.3656 | 4.18E-27 | 4.58E-26 | 50.63505 |
| TFF1 | 2.093536 | 8.500958 | 11.32423 | 6.14E-27 | 6.61E-26 | 50.25395 |
| CP | 1.580376 | 5.939927 | 11.31902 | 6.45E-27 | 6.91E-26 | 50.20599 |
| TCN1 | 1.861685 | 7.902029 | 11.29896 | 7.77E-27 | 8.26E-26 | 50.02142 |
| PNLIPRP1 | -3.11115 | 7.621003 | -11.2609 | 1.11E-26 | 1.16E-25 | 49.67214 |
| CCL18 | 1.605284 | 7.274036 | 11.19704 | 2.00E-26 | 2.06E-25 | 49.08675 |
| GATM | -1.61258 | 8.792774 | -11.0183 | 1.03E-25 | 1.00E-24 | 47.46105 |
| AMIGO2 | 1.555219 | 5.032001 | 10.97913 | 1.48E-25 | 1.41E-24 | 47.10673 |
| KCNJ16 | -1.58419 | 5.683341 | -10.824 | 6.05E-25 | 5.48E-24 | 45.7122 |
| AZGP1 | -1.54743 | 7.27672 | -10.5911 | 4.90E-24 | 4.14E-23 | 43.64408 |
| SYCN | -2.75805 | 8.325808 | -10.5219 | 9.06E-24 | 7.48E-23 | 43.03497 |
| GP2 | -2.87678 | 8.575074 | -10.3838 | 3.07E-23 | 2.43E-22 | 41.82812 |
| SCGN | -1.52406 | 6.483492 | -10.3525 | 4.05E-23 | 3.16E-22 | 41.55566 |
| CELA2B | -2.89036 | 8.139687 | -10.3162 | 5.56E-23 | 4.30E-22 | 41.24086 |
| PNLIPRP2 | -2.98768 | 8.615205 | -10.3112 | 5.81E-23 | 4.47E-22 | 41.19824 |
| SLC4A4 | -1.60777 | 7.721437 | -10.3041 | 6.18E-23 | 4.75E-22 | 41.13661 |
| ANXA10 | 1.742489 | 7.193599 | 10.12529 | 2.93E-22 | 2.12E-21 | 39.59793 |
| PRSS3 | -1.97781 | 9.119155 | -9.98663 | 9.69E-22 | 6.75E-21 | 38.41776 |
| DUOX2 | 1.585107 | 6.058907 | 9.947378 | 1.36E-21 | 9.30E-21 | 38.08574 |
| CTRC | -2.88419 | 8.755505 | -9.70809 | 1.03E-20 | 6.54E-20 | 36.08165 |
| CPA2 | -2.82257 | 9.100648 | -9.54918 | 3.89E-20 | 2.37E-19 | 34.76998 |
| CEL | -2.75956 | 9.105076 | -9.20953 | 6.33E-19 | 3.49E-18 | 32.01926 |
| CLPS | -2.77713 | 9.485825 | -9.20623 | 6.50E-19 | 3.58E-18 | 31.99288 |
| PLA2G1B | -2.6469 | 9.595577 | -8.89752 | 7.68E-18 | 3.88E-17 | 29.55746 |
| CELA3A | -2.50285 | 9.777091 | -8.60873 | 7.32E-17 | 3.37E-16 | 27.33608 |
| CPA1 | -2.56888 | 9.456473 | -8.33111 | 6.07E-16 | 2.60E-15 | 25.25378 |
| PNLIP | -2.32592 | 9.963364 | -7.63897 | 9.39E-14 | 3.37E-13 | 20.29811 |
| REG1B | -2.29583 | 9.166425 | -7.56163 | 1.62E-13 | 5.68E-13 | 19.76586 |
| REG3G | -1.72864 | 6.390606 | -7.54854 | 1.77E-13 | 6.19E-13 | 19.67628 |
| REG1A | -1.72787 | 10.54061 | -7.10459 | 3.64E-12 | 1.14E-11 | 16.7118 |
| CPB1 | -2.01062 | 10.18731 | -6.94655 | 1.03E-11 | 3.10E-11 | 15.69268 |
| REG3A | -1.74537 | 8.129425 | -6.78468 | 2.94E-11 | 8.50E-11 | 14.66893 |
