# Supplementary material for: AMIGO2 is a pivotal therapeutic target related to M2 polarization of macrophages in pancreatic ductal adenocarcinoma
Source: Aging (Albany NY). 2024 Jan 5;16(2):1111–27. doi: 10.18632/aging.205380 (PMC10866418; doi:10.18632/aging.205380)
Supplement: Supplementary Table 2 [file aging-16-205380-s002.docx]

Supplementary Table 2. Different expressed pathways between pancreatic cancer and normal tissue.

| ID | Description | setSize | enrichmentScore | NES | pvalue | p.adjust | qvalues | rank | leading_edge | core_enrichment |
| --- | --- | --- | --- | --- | --- | --- | --- | --- | --- | --- |
| KEGG_ECM_RECEPTOR_INTERACTION | KEGG_ECM_RECEPTOR_INTERACTION | 75 | 0.758443 | 2.488819 | 1.00E-10 | 1.80E-08 | 1.24E-08 | 1228 | tags=47%, list=9%, signal=43% | LAMC2/FN1/COL11A1/THBS2/COMP/LAMB3/COL1A1/COL3A1/COL5A2/ITGA2/COL5A1/COL6A3/LAMA3/ITGA3/SDC1/COL4A2/COL4A1/SPP1/ITGB5/ITGB4/HMMR/ITGA11/TNC/LAMA4/ITGA4/THBS1/IBSP/ITGA5/SDC4/COL6A2/ITGB8/HSPG2/LAMB1/CD47/LAMC1 |
| KEGG_FOCAL_ADHESION | KEGG_FOCAL_ADHESION | 170 | 0.578061 | 2.086181 | 3.67E-10 | 3.30E-08 | 2.28E-08 | 1771 | tags=35%, list=13%, signal=31% | LAMC2/FN1/COL11A1/THBS2/COMP/LAMB3/COL1A1/COL3A1/COL5A2/ITGA2/COL5A1/COL6A3/LAMA3/ITGA3/COL4A2/COL4A1/ACTB/SPP1/ITGB5/BIRC3/ITGB4/FLNA/ITGA11/PDGFRB/TNC/LAMA4/ACTN1/MYLK/PDGFC/MYL9/RAC2/ITGA4/THBS1/FLNB/IBSP/ITGA5/VEGFC/COL6A2/MYL12B/VAV1/VCL/ITGB8/MYL12A/CAPN2/CTNNB1/LAMB1/LAMC1/RAP1B/PPP1R12A/THBS3/PIK3CG/COL6A1/ITGA6/PAK1/ZYX/PDGFRA/LAMB2/PAK2/CAV1 |
| KEGG_MATURITY_ONSET_DIABETES_OF_THE_YOUNG | KEGG_MATURITY_ONSET_DIABETES_OF_THE_YOUNG | 22 | -0.83203 | -2.34498 | 7.88E-07 | 4.73E-05 | 3.26E-05 | 1630 | tags=77%, list=12%, signal=68% | HNF1A/GCK/PDX1/NEUROG3/NKX6-1/NKX2-2/PAX6/SLC2A2/HNF1B/NEUROD1/FOXA3/FOXA2/MNX1/HHEX/ONECUT1/NR5A2/IAPP |
| KEGG_PATHWAYS_IN_CANCER | KEGG_PATHWAYS_IN_CANCER | 268 | 0.457451 | 1.731194 | 1.26E-06 | 5.66E-05 | 3.91E-05 | 2247 | tags=30%, list=16%, signal=26% | LAMC2/FN1/LAMB3/ITGA2/SLC2A1/EGLN3/LEF1/LAMA3/ITGA3/PPARG/WNT2/MMP2/COL4A2/COL4A1/BIRC3/WNT5A/PDGFRB/BMP4/LAMA4/JUP/CDKN2B/FAS/RAC2/FZD7/GLI3/RUNX1T1/TGFBR1/HIF1A/RALA/E2F3/PIAS3/KITLG/VEGFC/CSF2RA/TGFB2/FZD1/CASP8/CTNNB1/LAMB1/MITF/CASP3/LAMC1/CTNNA1/GLI2/ETS1/CBLB/CKS1B/PML/RAD51/PIK3CG/RARB/RALB/KRAS/STK4/ITGA6/FGF1/CBLC/CBL/PDGFRA/HHIP/LAMB2/FZD2/HSP90AA1/BID/CCNE2/CDKN2A/KIT/LAMA2/RALBP1/FGF7/CCNE1/VEGFA/TGFA/TCF7L2/CDK6/GLI1/IKBKB/RBX1/ERBB2/TRAF3/FADD |
| KEGG_VALINE_LEUCINE_AND_ISOLEUCINE_DEGRADATION | KEGG_VALINE_LEUCINE_AND_ISOLEUCINE_DEGRADATION | 39 | -0.69937 | -2.26226 | 9.98E-06 | 0.000359 | 0.000248 | 2365 | tags=69%, list=17%, signal=58% | EHHADH/ACAA2/ACAA1/DLD/HADHB/ACAD8/ALDH9A1/BCKDHB/PCCA/BCKDHA/HADHA/HIBCH/PCCB/AUH/ACADS/HADH/MCEE/ACADSB/BCAT2/ACADM/MCCC1/ALDH6A1/ALDH3A2/BCAT1/ACAT1/ABAT/AOX1 |
| KEGG_SMALL_CELL_LUNG_CANCER | KEGG_SMALL_CELL_LUNG_CANCER | 72 | 0.603574 | 1.971394 | 1.59E-05 | 0.000478 | 0.00033 | 2246 | tags=36%, list=16%, signal=30% | LAMC2/FN1/LAMB3/ITGA2/LAMA3/ITGA3/COL4A2/COL4A1/BIRC3/LAMA4/CDKN2B/E2F3/PIAS3/LAMB1/LAMC1/CKS1B/PIK3CG/RARB/ITGA6/LAMB2/CCNE2/LAMA2/CCNE1/CDK6/IKBKB/TRAF3 |
| KEGG_GLYCINE_SERINE_AND_THREONINE_METABOLISM | KEGG_GLYCINE_SERINE_AND_THREONINE_METABOLISM | 28 | -0.73504 | -2.22291 | 2.76E-05 | 0.000671 | 0.000463 | 575 | tags=43%, list=4%, signal=41% | SHMT2/SRR/CHDH/SARDH/MAOA/CTH/PHGDH/GCAT/GAMT/PSAT1/GATM/GNMT |
| KEGG_VIRAL_MYOCARDITIS | KEGG_VIRAL_MYOCARDITIS | 54 | 0.628965 | 1.953624 | 2.98E-05 | 0.000671 | 0.000463 | 2155 | tags=44%, list=15%, signal=38% | CD55/ACTB/HLA-DQA1/HLA-DPA1/ITGB2/HLA-F/RAC2/HLA-DMB/CD86/SGCD/SGCB/CASP8/CASP3/HLA-DMA/HLA-DQB1/MYH9/HLA-DPB1/MYH10/CD80/BID/CAV1/LAMA2/MYH11/EIF4G3 |
| KEGG_LEISHMANIA_INFECTION | KEGG_LEISHMANIA_INFECTION | 43 | 0.6638 | 1.956991 | 4.30E-05 | 0.000859 | 0.000593 | 1676 | tags=44%, list=12%, signal=39% | NCF2/FCGR2A/HLA-DQA1/HLA-DPA1/ITGB2/ITGA4/HLA-DMB/C3/TGFB2/IFNGR2/JAK2/HLA-DMA/HLA-DQB1/MARCKSL1/HLA-DPB1/MYD88/NCF4/IFNGR1/TLR4 |
| KEGG_CELL_CYCLE | KEGG_CELL_CYCLE | 101 | 0.540867 | 1.829066 | 4.81E-05 | 0.000866 | 0.000598 | 2369 | tags=38%, list=17%, signal=31% | SFN/CCNB1/CCNA2/CCNB2/TTK/BUB1/CDKN2B/MAD2L1/CDC6/CDC20/CHEK1/E2F3/TGFB2/YWHAZ/MCM6/CDC7/CDC25B/MCM2/MCM4/GADD45A/MCM3/CDK7/MAD1L1/BUB3/RAD21/WEE1/CDC45/CCNE2/CDKN2A/CHEK2/CDC23/CCNE1/CDK6/PCNA/RBX1/DBF4/ATR/CDC25C |
| KEGG_PATHOGENIC_ESCHERICHIA_COLI_INFECTION | KEGG_PATHOGENIC_ESCHERICHIA_COLI_INFECTION | 39 | 0.669695 | 1.933511 | 5.30E-05 | 0.000867 | 0.000598 | 1942 | tags=51%, list=14%, signal=44% | ACTB/LY96/TUBA1C/TUBA4A/EZR/HCLS1/CD14/YWHAZ/TUBB/CLDN1/CTTN/NCK1/CTNNB1/TUBA1B/KRT18/TLR4/TUBA1A/ARPC5/ARPC3/ARPC5L |
| KEGG_ALLOGRAFT_REJECTION | KEGG_ALLOGRAFT_REJECTION | 20 | 0.767988 | 1.949762 | 7.10E-05 | 0.001065 | 0.000735 | 1724 | tags=50%, list=12%, signal=44% | HLA-DQA1/HLA-DPA1/FAS/HLA-F/HLA-DMB/CD86/HLA-DMA/HLA-DQB1/HLA-DPB1/CD80 |
| KEGG_GRAFT_VERSUS_HOST_DISEASE | KEGG_GRAFT_VERSUS_HOST_DISEASE | 17 | 0.782396 | 1.916568 | 0.000152 | 0.001916 | 0.001322 | 1724 | tags=59%, list=12%, signal=52% | HLA-DQA1/HLA-DPA1/FAS/HLA-F/HLA-DMB/CD86/HLA-DMA/HLA-DQB1/HLA-DPB1/CD80 |
| KEGG_CELL_ADHESION_MOLECULES_CAMS | KEGG_CELL_ADHESION_MOLECULES_CAMS | 110 | 0.509442 | 1.748822 | 0.000155 | 0.001916 | 0.001322 | 2138 | tags=34%, list=15%, signal=29% | CLDN18/VCAN/CDH3/SDC1/HLA-DQA1/CDH2/CD58/HLA-DPA1/ITGB2/HLA-F/ITGA4/PTPRC/CLDN2/HLA-DMB/CLDN23/CLDN4/CD86/SDC4/CLDN11/CLDN1/ITGB8/SELL/JAM3/HLA-DMA/HLA-DQB1/MPZL1/HLA-DPB1/CD276/CD2/ITGA6/CD80/CNTN1/PDCD1LG2/CTLA4/SELPLG/CD274/VCAM1 |
| KEGG_LEUKOCYTE_TRANSENDOTHELIAL_MIGRATION | KEGG_LEUKOCYTE_TRANSENDOTHELIAL_MIGRATION | 95 | 0.533006 | 1.785212 | 0.00016 | 0.001916 | 0.001322 | 1466 | tags=32%, list=11%, signal=28% | CLDN18/NCF2/THY1/MMP2/ACTB/ACTN1/MSN/ITGB2/MYL9/CYBB/RAC2/ITGA4/CLDN2/CLDN23/EZR/CLDN4/CLDN11/MYL12B/GNAI2/CLDN1/VAV1/VCL/MYL12A/RHOH/CTNNB1/JAM3/CTNNA1/RAP1B/ITK/PIK3CG |
| KEGG_ARRHYTHMOGENIC_RIGHT_VENTRICULAR_CARDIOMYOPATHY_ARVC | KEGG_ARRHYTHMOGENIC_RIGHT_VENTRICULAR_CARDIOMYOPATHY_ARVC | 67 | 0.559459 | 1.792718 | 0.000255 | 0.002872 | 0.001982 | 2278 | tags=42%, list=16%, signal=35% | ITGA2/LEF1/ITGA3/ACTB/ITGB5/ITGB4/ITGA11/CDH2/JUP/ACTN1/ITGA4/DSG2/GJA1/ITGA5/SGCD/SGCB/ITGB8/CACNB3/CTNNB1/CTNNA1/DSC2/ITGA6/LAMA2/ATP2A2/TCF7L2/SLC8A1/ITGB3/CACNA2D1 |
| KEGG_PROPANOATE_METABOLISM | KEGG_PROPANOATE_METABOLISM | 29 | -0.67658 | -2.05718 | 0.000333 | 0.003522 | 0.002431 | 1632 | tags=55%, list=12%, signal=49% | ALDH9A1/PCCA/HADHA/HIBCH/PCCB/SUCLG1/MCEE/LDHB/ACADM/MLYCD/ALDH6A1/ALDH3A2/ACSS1/ACACB/ACAT1/ABAT |
| KEGG_CYTOKINE_CYTOKINE_RECEPTOR_INTERACTION | KEGG_CYTOKINE_CYTOKINE_RECEPTOR_INTERACTION | 208 | 0.431371 | 1.596637 | 0.000395 | 0.003953 | 0.002728 | 2239 | tags=29%, list=16%, signal=25% | CXCL5/CCL20/CCL18/CXCL10/IL1R2/TNFSF4/IL2RG/IL1RAP/LIF/CXCL9/TNFRSF21/IL20RB/PDGFRB/CXCL14/TNFSF13B/IL7R/ACVR1/PDGFC/FAS/CXCL3/OSMR/TNFRSF10A/TNFSF11/CCL13/IL7/IL2RA/TGFBR1/BMPR2/KITLG/VEGFC/CSF2RA/TGFB2/TNFSF10/CCL19/CXCL16/TNFSF15/IFNGR2/IL4R/CCR1/IFNE/IL13RA1/TNFRSF10D/TNFRSF9/IL10RA/TNFRSF11A/TNFRSF10B/PDGFRA/IFNGR1/PLEKHO2/IL18R1/KIT/CCR6/CCL5/VEGFA/IL15/IFNAR2/IL2RB/CCR7/TNFRSF1B/CCL26 |
| KEGG_AUTOIMMUNE_THYROID_DISEASE | KEGG_AUTOIMMUNE_THYROID_DISEASE | 27 | 0.694289 | 1.885283 | 0.000461 | 0.004363 | 0.00301 | 1948 | tags=41%, list=14%, signal=35% | HLA-DQA1/HLA-DPA1/FAS/HLA-F/HLA-DMB/CD86/HLA-DMA/HLA-DQB1/HLA-DPB1/CD80/CTLA4 |
| KEGG_PROTEIN_EXPORT | KEGG_PROTEIN_EXPORT | 21 | -0.72345 | -2.00958 | 0.000636 | 0.00572 | 0.003947 | 2581 | tags=71%, list=19%, signal=58% | IMMP1L/IMMP2L/SRP54/SPCS2/SRP19/SRP72/SEC62/OXA1L/SPCS1/SPCS3/SEC61B/SEC61A1/SEC63/SRPRB/SEC11C |
| KEGG_O_GLYCAN_BIOSYNTHESIS | KEGG_O_GLYCAN_BIOSYNTHESIS | 24 | 0.703112 | 1.848709 | 0.000973 | 0.008337 | 0.005753 | 2065 | tags=50%, list=15%, signal=43% | GCNT3/GALNT5/ST6GALNAC1/C1GALT1/GALNT10/B4GALT5/GCNT1/GALNT3/ST3GAL1/GALNT6/GALNT14/GALNT12 |
| KEGG_ANTIGEN_PROCESSING_AND_PRESENTATION | KEGG_ANTIGEN_PROCESSING_AND_PRESENTATION | 45 | 0.600325 | 1.787976 | 0.001046 | 0.008561 | 0.005908 | 1733 | tags=33%, list=12%, signal=29% | CTSB/CTSS/HLA-DQA1/HLA-DPA1/HLA-F/CD74/HLA-DMB/TAP2/HSPA6/HLA-DMA/HLA-DQB1/LGMN/HLA-DPB1/RFX5/HSP90AA1 |
| KEGG_P53_SIGNALING_PATHWAY | KEGG_P53_SIGNALING_PATHWAY | 58 | 0.554301 | 1.747043 | 0.001158 | 0.00906 | 0.006252 | 2762 | tags=50%, list=20%, signal=40% | SERPINB5/SFN/CCNB1/PMAIP1/CCNB2/FAS/RRM2/THBS1/CHEK1/PERP/CASP8/CASP3/GADD45A/TNFRSF10B/BID/CCNE2/CDKN2A/CHEK2/CCNG2/CCNE1/CDK6/SHISA5/ATR/CD82/TP53I3/SESN3/BAX/APAF1/GTSE1 |
| KEGG_RIBOSOME | KEGG_RIBOSOME | 58 | -0.53843 | -1.83191 | 0.001605 | 0.012039 | 0.008307 | 2741 | tags=45%, list=20%, signal=36% | UBA52/RPL31/RPS6/RPL35A/RPL22L1/RPL18/RPL3/RPL38/RPL29/RPL7/RPS23/RPS29/RPS15/RPS5/RPL14/RPS9/RPL36AL/RPL22/RPL27A/RSL24D1/RPLP2/RPL36/RPL15/RPL10L/RPS4Y1/RPL3L |
| KEGG_PROTEASOME | KEGG_PROTEASOME | 36 | 0.603925 | 1.711223 | 0.003174 | 0.021925 | 0.015129 | 4360 | tags=75%, list=31%, signal=52% | PSMB9/PSMB8/PSMD1/PSMD14/PSMA5/PSMC4/PSMB3/PSMA4/PSMD2/PSMD11/PSMC2/PSMA1/PSMA3/PSMA7/PSME4/PSMD13/POMP/PSMB4/PSME1/PSMC6/PSMA2/PSMD4/PSMD8/PSMC1/PSME3/PSMD3/PSMB2 |
| KEGG_TOLL_LIKE_RECEPTOR_SIGNALING_PATHWAY | KEGG_TOLL_LIKE_RECEPTOR_SIGNALING_PATHWAY | 78 | 0.489816 | 1.615913 | 0.003226 | 0.021925 | 0.015129 | 2389 | tags=31%, list=17%, signal=26% | CTSK/CXCL10/SPP1/CXCL9/LY96/CD86/CD14/MAP3K8/CASP8/TBK1/TLR7/PIK3CG/MYD88/IRF7/TLR4/CD80/TLR8/CCL5/IFNAR2/IKBKB/TLR6/TRAF3/FADD/PIK3CA |
| KEGG_TYROSINE_METABOLISM | KEGG_TYROSINE_METABOLISM | 35 | -0.5844 | -1.84031 | 0.003289 | 0.021925 | 0.015129 | 997 | tags=23%, list=7%, signal=21% | GSTZ1/ADH1A/ADH1C/HGD/MAOA/DDC/ADH1B/AOX1 |
| KEGG_PROXIMAL_TUBULE_BICARBONATE_RECLAMATION | KEGG_PROXIMAL_TUBULE_BICARBONATE_RECLAMATION | 20 | -0.69033 | -1.89687 | 0.003671 | 0.0236 | 0.016285 | 1853 | tags=55%, list=13%, signal=48% | ATP1A2/PCK1/ATP1A1/SLC25A10/ATP1B2/PCK2/SLC38A3/AQP1/GLS2/CA4/SLC4A4 |
| KEGG_FC_GAMMA_R_MEDIATED_PHAGOCYTOSIS | KEGG_FC_GAMMA_R_MEDIATED_PHAGOCYTOSIS | 70 | 0.49232 | 1.600708 | 0.004583 | 0.028446 | 0.01963 | 2871 | tags=43%, list=21%, signal=34% | ASAP2/FCGR2A/FCGR2B/SCIN/RAC2/ASAP1/PTPRC/PLA2G4A/VAV1/DOCK2/CFL1/MARCKSL1/HCK/PIK3CG/PAK1/DNM1L/SPHK1/ARPC5/ARPC3/DNM3/GSN/ARPC5L/INPP5D/ARF6/PIK3CA/AKT3/WASF1/DNM1/MAP2K1/PRKCB |
| KEGG_NATURAL_KILLER_CELL_MEDIATED_CYTOTOXICITY | KEGG_NATURAL_KILLER_CELL_MEDIATED_CYTOTOXICITY | 83 | 0.477025 | 1.58143 | 0.005915 | 0.035277 | 0.024343 | 3430 | tags=45%, list=25%, signal=34% | FCER1G/ITGB2/FAS/RAC2/TNFRSF10A/TYROBP/LCP2/TNFSF10/VAV1/IFNGR2/LCK/CASP3/TNFRSF10D/PPP3CA/PIK3CG/KRAS/TNFRSF10B/PAK1/IFNGR1/BID/IFNAR2/MICA/PPP3R1/ULBP2/PIK3CA/ITGAL/SHC4/CD48/MAP2K1/PRKCB/SHC1/PRKCA/SH2D1A/NFAT5/GRB2/CD247/SH3BP2 |
| KEGG_REGULATION_OF_ACTIN_CYTOSKELETON | KEGG_REGULATION_OF_ACTIN_CYTOSKELETON | 181 | 0.407613 | 1.484072 | 0.006075 | 0.035277 | 0.024343 | 2846 | tags=38%, list=20%, signal=31% | FN1/ITGA2/ITGA3/ACTB/ITGB5/ITGB4/ITGA11/PDGFRB/SCIN/F2R/ACTN1/RRAS/MYLK/MSN/ITGB2/PDGFC/MYL9/RAC2/ITGA4/EZR/ITGA5/CD14/IQGAP1/MYL12B/VAV1/VCL/ITGB8/MYL12A/NCKAP1L/DIAPH3/IQGAP3/MYH9/CFL1/SSH1/PPP1R12A/PIK3CG/KRAS/MYH10/ITGA6/FGF1/PAK1/PDGFRA/ITGAX/PAK2/GNA13/ARPC5/TIAM2/ARPC3/PFN1/GSN/FGF7/ARPC5L/DIAPH2/PIP4K2A/TIAM1/MRAS/ITGB3/ARHGEF6/PIK3CA/ABI2/PIP4K2C/ITGAL/FGF2/SLC9A1/WASF1/ITGA9/MYH14/MAP2K1/FGF19 |
| KEGG_LYSOSOME | KEGG_LYSOSOME | 106 | 0.453397 | 1.544421 | 0.00643 | 0.036166 | 0.024957 | 3221 | tags=40%, list=23%, signal=31% | CTSK/CTSB/LAPTM5/CTSS/AP1S3/ACP5/AP3M2/CTSC/CTSA/CLTB/NAGA/CTSD/LGMN/GLA/SLC11A1/GNS/IDS/GNPTAB/CTSZ/PLA2G15/GALNS/ATP6V0D2/MANBA/PPT1/LAPTM4A/CTSO/AP1M1/GALC/PSAP/SLC17A5/AP4E1/IGF2R/CTSH/M6PR/HEXA/CD63/ATP6V1H/GM2A/LAPTM4B/ATP6V0C/CLTC/AP1S1 |
| KEGG_FATTY_ACID_METABOLISM | KEGG_FATTY_ACID_METABOLISM | 34 | -0.56476 | -1.76578 | 0.007778 | 0.041767 | 0.028822 | 2365 | tags=59%, list=17%, signal=49% | EHHADH/ACAA2/ACAA1/HADHB/ALDH9A1/HADHA/ACOX3/CPT2/ACOX1/ACADS/HADH/ACADSB/ADH1A/ACADVL/ACADM/ADH1C/ALDH3A2/ACAT1/ADH1B/ACADL |
| KEGG_ASTHMA | KEGG_ASTHMA | 21 | 0.659142 | 1.688341 | 0.007889 | 0.041767 | 0.028822 | 2768 | tags=48%, list=20%, signal=38% | FCER1G/HLA-DQA1/HLA-DPA1/HLA-DMB/HLA-DMA/HLA-DQB1/HLA-DPB1/MS4A2/HLA-DOB/CCL11 |
| KEGG_N_GLYCAN_BIOSYNTHESIS | KEGG_N_GLYCAN_BIOSYNTHESIS | 38 | -0.53653 | -1.7154 | 0.008213 | 0.042238 | 0.029147 | 2548 | tags=42%, list=18%, signal=34% | ALG12/DPAGT1/RFT1/DPM2/B4GALT2/MAN2A1/ALG9/TUSC3/DPM3/ALG5/ALG2/MAN2A2/MAN1A1/MAN1A2/ALG14/MGAT4A |
