# Supplementary material for: AMIGO2 is a pivotal therapeutic target related to M2 polarization of macrophages in pancreatic ductal adenocarcinoma
Source: Aging (Albany NY). 2024 Jan 5;16(2):1111–27. doi: 10.18632/aging.205380 (PMC10866418; doi:10.18632/aging.205380)
Supplement: Supplementary Tables 3 and 4 [file aging-16-205380-s003.pdf]

SUPPLEMENTARY TABLES

Supplementary Table 3. The result of LASSO regression in combined GEO datasets.

SULF1
LAMC2
COL10A1
AHNAK2
POSTN
NOX4
S100P
ITGA2
IFI27
SLC6A14
NQO1
COL5A2
SLPI
GALNT5
GREM1
P2RX1
MMP11
CXCL5
COL17A1
TNFAIP6
AMIGO2
PI3
NRG4
PSAT1
LCN2
IAPP
FGL1
BEX1
TFF1
AZGP1
SYCN
DUOX2
REG3A

Supplementary Table 4. The result of SVM-RFE regression in combined GEO datasets.

COL10A1
S100P
LAMC2
SLC6A14
SULF1
AHNAK2
SERPINB5
SFN
IFI27
FN1
TMPRSS4
POSTN
AMIGO2
