# Supplementary material for: AMIGO2 is a pivotal therapeutic target related to M2 polarization of macrophages in pancreatic ductal adenocarcinoma
Source: Aging (Albany NY). 2024 Jan 5;16(2):1111–27. doi: 10.18632/aging.205380 (PMC10866418; doi:10.18632/aging.205380)
Supplement: Supplementary Table 5 [file aging-16-205380-s004.docx]

Supplementary Table 5. The result of GSEA in combined GEO datasets.

| NAME | GS<br> follow link to MSigDB | GS DETAILS | SIZE | ES | NES | NOM p-val | FDR q-val | FWER p-val | RANK AT MAX | LEADING EDGE |
| --- | --- | --- | --- | --- | --- | --- | --- | --- | --- | --- |
| KEGG_P53_SIGNALING_PATHWAY | KEGG_P53_SIGNALING_PATHWAY | Details ... | 68 | 0.594708 | 1.847092 | 0 | 0.200296 | 0.104 | 4757 | tags=49%, list=15%, signal=57% |
| KEGG_SMALL_CELL_LUNG_CANCER | KEGG_SMALL_CELL_LUNG_CANCER | Details ... | 84 | 0.585694 | 1.792402 | 0.004049 | 0.189415 | 0.181 | 4055 | tags=42%, list=12%, signal=47% |
| KEGG_CELL_CYCLE | KEGG_CELL_CYCLE | Details ... | 123 | 0.590693 | 1.76647 | 0.009881 | 0.170164 | 0.223 | 5905 | tags=50%, list=18%, signal=60% |
| KEGG_THYROID_CANCER | KEGG_THYROID_CANCER | Details ... | 29 | 0.554817 | 1.756537 | 0.004149 | 0.141776 | 0.236 | 4375 | tags=41%, list=13%, signal=48% |
| KEGG_GLYCOSAMINOGLYCAN_BIOSYNTHESIS_CHONDROITIN_SULFATE | KEGG_GLYCOSAMINOGLYCAN_BIOSYNTHESIS_CHONDROITIN_SULFATE | Details ... | 22 | 0.690303 | 1.747545 | 0.010661 | 0.127167 | 0.252 | 5746 | tags=59%, list=18%, signal=72% |
| KEGG_PROTEASOME | KEGG_PROTEASOME | Details ... | 46 | 0.580215 | 1.74384 | 0.025194 | 0.110725 | 0.26 | 10912 | tags=78%, list=33%, signal=117% |
| KEGG_PATHWAYS_IN_CANCER | KEGG_PATHWAYS_IN_CANCER | Details ... | 324 | 0.501593 | 1.734333 | 0.00431 | 0.105895 | 0.284 | 4101 | tags=35%, list=13%, signal=39% |
| KEGG_CHRONIC_MYELOID_LEUKEMIA | KEGG_CHRONIC_MYELOID_LEUKEMIA | Details ... | 72 | 0.544308 | 1.731781 | 0.010288 | 0.095028 | 0.286 | 5926 | tags=42%, list=18%, signal=51% |
| KEGG_BASAL_CELL_CARCINOMA | KEGG_BASAL_CELL_CARCINOMA | Details ... | 55 | 0.570556 | 1.717097 | 0.018828 | 0.097859 | 0.311 | 4338 | tags=42%, list=13%, signal=48% |
| KEGG_BLADDER_CANCER | KEGG_BLADDER_CANCER | Details ... | 42 | 0.552707 | 1.706411 | 0.016162 | 0.100305 | 0.335 | 4395 | tags=48%, list=13%, signal=55% |
| KEGG_FOCAL_ADHESION | KEGG_FOCAL_ADHESION | Details ... | 199 | 0.547764 | 1.704696 | 0.028761 | 0.092626 | 0.339 | 5064 | tags=49%, list=15%, signal=58% |
| KEGG_AXON_GUIDANCE | KEGG_AXON_GUIDANCE | Details ... | 129 | 0.504411 | 1.700573 | 0.004283 | 0.089169 | 0.352 | 5089 | tags=40%, list=16%, signal=47% |
| KEGG_ECM_RECEPTOR_INTERACTION | KEGG_ECM_RECEPTOR_INTERACTION | Details ... | 84 | 0.628407 | 1.68005 | 0.032538 | 0.100515 | 0.39 | 3870 | tags=56%, list=12%, signal=63% |
| KEGG_NOTCH_SIGNALING_PATHWAY | KEGG_NOTCH_SIGNALING_PATHWAY | Details ... | 47 | 0.516339 | 1.679001 | 0.012658 | 0.093774 | 0.392 | 5557 | tags=43%, list=17%, signal=51% |
| KEGG_PANCREATIC_CANCER | KEGG_PANCREATIC_CANCER | Details ... | 69 | 0.537548 | 1.674343 | 0.018908 | 0.092555 | 0.405 | 5731 | tags=48%, list=18%, signal=58% |
| KEGG_SPLICEOSOME | KEGG_SPLICEOSOME | Details ... | 127 | 0.489297 | 1.674183 | 0.047801 | 0.086992 | 0.405 | 11518 | tags=65%, list=35%, signal=100% |
| KEGG_TIGHT_JUNCTION | KEGG_TIGHT_JUNCTION | Details ... | 132 | 0.453092 | 1.659042 | 0.008163 | 0.094184 | 0.441 | 7174 | tags=48%, list=22%, signal=61% |
| KEGG_PATHOGENIC_ESCHERICHIA_COLI_INFECTION | KEGG_PATHOGENIC_ESCHERICHIA_COLI_INFECTION | Details ... | 56 | 0.496186 | 1.65738 | 0.01848 | 0.090312 | 0.445 | 8531 | tags=59%, list=26%, signal=80% |
| KEGG_NUCLEOTIDE_EXCISION_REPAIR | KEGG_NUCLEOTIDE_EXCISION_REPAIR | Details ... | 44 | 0.540163 | 1.657333 | 0.036468 | 0.085559 | 0.445 | 9328 | tags=64%, list=29%, signal=89% |
| KEGG_ADHERENS_JUNCTION | KEGG_ADHERENS_JUNCTION | Details ... | 73 | 0.518943 | 1.649859 | 0.023355 | 0.08719 | 0.462 | 5644 | tags=49%, list=17%, signal=59% |
| KEGG_RENAL_CELL_CARCINOMA | KEGG_RENAL_CELL_CARCINOMA | Details ... | 69 | 0.510169 | 1.644866 | 0.03397 | 0.087022 | 0.474 | 5064 | tags=41%, list=15%, signal=48% |
| KEGG_HEDGEHOG_SIGNALING_PATHWAY | KEGG_HEDGEHOG_SIGNALING_PATHWAY | Details ... | 56 | 0.537899 | 1.644504 | 0.014831 | 0.083524 | 0.474 | 4338 | tags=38%, list=13%, signal=43% |
| KEGG_BASAL_TRANSCRIPTION_FACTORS | KEGG_BASAL_TRANSCRIPTION_FACTORS | Details ... | 35 | 0.530563 | 1.617396 | 0.017893 | 0.100745 | 0.526 | 6933 | tags=49%, list=21%, signal=62% |
| KEGG_REGULATION_OF_ACTIN_CYTOSKELETON | KEGG_REGULATION_OF_ACTIN_CYTOSKELETON | Details ... | 213 | 0.461025 | 1.613603 | 0.026667 | 0.099983 | 0.53 | 5657 | tags=42%, list=17%, signal=51% |
| KEGG_WNT_SIGNALING_PATHWAY | KEGG_WNT_SIGNALING_PATHWAY | Details ... | 150 | 0.446482 | 1.612714 | 0.017094 | 0.096871 | 0.535 | 5300 | tags=37%, list=16%, signal=44% |
| KEGG_GLIOMA | KEGG_GLIOMA | Details ... | 65 | 0.482166 | 1.582342 | 0.023605 | 0.118221 | 0.601 | 6009 | tags=42%, list=18%, signal=51% |
| KEGG_ARRHYTHMOGENIC_RIGHT_VENTRICULAR_CARDIOMYOPATHY_ARVC | KEGG_ARRHYTHMOGENIC_RIGHT_VENTRICULAR_CARDIOMYOPATHY_ARVC | Details ... | 74 | 0.502127 | 1.574626 | 0.032609 | 0.11996 | 0.614 | 1768 | tags=31%, list=5%, signal=33% |
| KEGG_PROSTATE_CANCER | KEGG_PROSTATE_CANCER | Details ... | 89 | 0.497469 | 1.570283 | 0.055202 | 0.11929 | 0.623 | 6009 | tags=36%, list=18%, signal=44% |
| KEGG_TGF_BETA_SIGNALING_PATHWAY | KEGG_TGF_BETA_SIGNALING_PATHWAY | Details ... | 84 | 0.509186 | 1.528858 | 0.07234 | 0.15587 | 0.699 | 5571 | tags=36%, list=17%, signal=43% |
| KEGG_BIOSYNTHESIS_OF_UNSATURATED_FATTY_ACIDS | KEGG_BIOSYNTHESIS_OF_UNSATURATED_FATTY_ACIDS | Details ... | 22 | 0.546275 | 1.521172 | 0.057447 | 0.159656 | 0.724 | 7433 | tags=59%, list=23%, signal=76% |
| KEGG_ENDOMETRIAL_CANCER | KEGG_ENDOMETRIAL_CANCER | Details ... | 52 | 0.47762 | 1.507786 | 0.052174 | 0.1689 | 0.741 | 4796 | tags=33%, list=15%, signal=38% |
| KEGG_PYRIMIDINE_METABOLISM | KEGG_PYRIMIDINE_METABOLISM | Details ... | 98 | 0.445205 | 1.50321 | 0.09144 | 0.168491 | 0.752 | 8600 | tags=45%, list=26%, signal=61% |
| KEGG_COLORECTAL_CANCER | KEGG_COLORECTAL_CANCER | Details ... | 61 | 0.466662 | 1.501805 | 0.060086 | 0.164968 | 0.754 | 7057 | tags=43%, list=22%, signal=54% |
| KEGG_MELANOGENESIS | KEGG_MELANOGENESIS | Details ... | 101 | 0.427863 | 1.496041 | 0.041037 | 0.166174 | 0.761 | 3340 | tags=28%, list=10%, signal=31% |
| KEGG_ERBB_SIGNALING_PATHWAY | KEGG_ERBB_SIGNALING_PATHWAY | Details ... | 87 | 0.42045 | 1.486881 | 0.080082 | 0.170533 | 0.774 | 7057 | tags=43%, list=22%, signal=54% |
| KEGG_GLYCOSAMINOGLYCAN_BIOSYNTHESIS_KERATAN_SULFATE | KEGG_GLYCOSAMINOGLYCAN_BIOSYNTHESIS_KERATAN_SULFATE | Details ... | 15 | 0.554028 | 1.4805 | 0.072765 | 0.172454 | 0.782 | 3789 | tags=40%, list=12%, signal=45% |
| KEGG_DNA_REPLICATION | KEGG_DNA_REPLICATION | Details ... | 36 | 0.625297 | 1.478325 | 0.099222 | 0.170314 | 0.79 | 8444 | tags=69%, list=26%, signal=93% |
| KEGG_MISMATCH_REPAIR | KEGG_MISMATCH_REPAIR | Details ... | 23 | 0.591746 | 1.470441 | 0.100394 | 0.17407 | 0.804 | 7235 | tags=52%, list=22%, signal=67% |
| KEGG_MELANOMA | KEGG_MELANOMA | Details ... | 71 | 0.447853 | 1.469192 | 0.05531 | 0.170885 | 0.805 | 5392 | tags=38%, list=16%, signal=45% |
| KEGG_GLYCOSPHINGOLIPID_BIOSYNTHESIS_LACTO_AND_NEOLACTO_SERIES | KEGG_GLYCOSPHINGOLIPID_BIOSYNTHESIS_LACTO_AND_NEOLACTO_SERIES | Details ... | 26 | 0.481518 | 1.45649 | 0.038685 | 0.179726 | 0.819 | 4199 | tags=46%, list=13%, signal=53% |
| KEGG_HYPERTROPHIC_CARDIOMYOPATHY_HCM | KEGG_HYPERTROPHIC_CARDIOMYOPATHY_HCM | Details ... | 82 | 0.448307 | 1.446571 | 0.084567 | 0.185746 | 0.83 | 2664 | tags=26%, list=8%, signal=28% |
| KEGG_OOCYTE_MEIOSIS | KEGG_OOCYTE_MEIOSIS | Details ... | 112 | 0.394382 | 1.437046 | 0.082495 | 0.191641 | 0.841 | 6317 | tags=35%, list=19%, signal=43% |
| KEGG_PENTOSE_PHOSPHATE_PATHWAY | KEGG_PENTOSE_PHOSPHATE_PATHWAY | Details ... | 26 | 0.451449 | 1.420539 | 0.108365 | 0.204221 | 0.857 | 8715 | tags=58%, list=27%, signal=79% |
| KEGG_NEUROTROPHIN_SIGNALING_PATHWAY | KEGG_NEUROTROPHIN_SIGNALING_PATHWAY | Details ... | 126 | 0.397437 | 1.401692 | 0.124197 | 0.220565 | 0.87 | 7270 | tags=41%, list=22%, signal=53% |
| KEGG_LYSOSOME | KEGG_LYSOSOME | Details ... | 121 | 0.388439 | 1.397047 | 0.134576 | 0.220787 | 0.876 | 8655 | tags=41%, list=26%, signal=56% |
| KEGG_LEUKOCYTE_TRANSENDOTHELIAL_MIGRATION | KEGG_LEUKOCYTE_TRANSENDOTHELIAL_MIGRATION | Details ... | 116 | 0.44415 | 1.395372 | 0.14135 | 0.217769 | 0.877 | 6002 | tags=45%, list=18%, signal=55% |
| KEGG_BASE_EXCISION_REPAIR | KEGG_BASE_EXCISION_REPAIR | Details ... | 35 | 0.526693 | 1.387228 | 0.133072 | 0.221652 | 0.882 | 7751 | tags=60%, list=24%, signal=79% |
| KEGG_ENDOCYTOSIS | KEGG_ENDOCYTOSIS | Details ... | 181 | 0.366539 | 1.364404 | 0.139004 | 0.244044 | 0.903 | 8297 | tags=46%, list=25%, signal=62% |
| KEGG_VEGF_SIGNALING_PATHWAY | KEGG_VEGF_SIGNALING_PATHWAY | Details ... | 76 | 0.384515 | 1.363067 | 0.140167 | 0.240288 | 0.904 | 6273 | tags=37%, list=19%, signal=45% |
| KEGG_GALACTOSE_METABOLISM | KEGG_GALACTOSE_METABOLISM | Details ... | 26 | 0.449488 | 1.359722 | 0.118577 | 0.239158 | 0.905 | 3948 | tags=38%, list=12%, signal=44% |
| KEGG_UBIQUITIN_MEDIATED_PROTEOLYSIS | KEGG_UBIQUITIN_MEDIATED_PROTEOLYSIS | | 134 | 0.377422 | 1.35164 | 0.196393 | 0.243884 | 0.908 | 8327 | tags=41%, list=25%, signal=55% |
| KEGG_DILATED_CARDIOMYOPATHY | KEGG_DILATED_CARDIOMYOPATHY | | 89 | 0.418625 | 1.33878 | 0.151844 | 0.254677 | 0.915 | 2664 | tags=25%, list=8%, signal=27% |
| KEGG_DRUG_METABOLISM_OTHER_ENZYMES | KEGG_DRUG_METABOLISM_OTHER_ENZYMES | | 51 | 0.455708 | 1.3356 | 0.11359 | 0.253648 | 0.918 | 4440 | tags=33%, list=14%, signal=39% |
| KEGG_NON_SMALL_CELL_LUNG_CANCER | KEGG_NON_SMALL_CELL_LUNG_CANCER | | 54 | 0.430498 | 1.334242 | 0.186335 | 0.25122 | 0.92 | 4796 | tags=30%, list=15%, signal=35% |
| KEGG_GLYCOLYSIS_GLUCONEOGENESIS | KEGG_GLYCOLYSIS_GLUCONEOGENESIS | | 62 | 0.390005 | 1.316214 | 0.1409 | 0.26716 | 0.926 | 3787 | tags=34%, list=12%, signal=38% |
| KEGG_HOMOLOGOUS_RECOMBINATION | KEGG_HOMOLOGOUS_RECOMBINATION | | 28 | 0.521529 | 1.290695 | 0.241715 | 0.293083 | 0.936 | 7771 | tags=57%, list=24%, signal=75% |
| KEGG_RIG_I_LIKE_RECEPTOR_SIGNALING_PATHWAY | KEGG_RIG_I_LIKE_RECEPTOR_SIGNALING_PATHWAY | | 70 | 0.38875 | 1.2739 | 0.202899 | 0.308118 | 0.946 | 8075 | tags=43%, list=25%, signal=57% |
| KEGG_GAP_JUNCTION | KEGG_GAP_JUNCTION | | 90 | 0.374013 | 1.268421 | 0.183158 | 0.309722 | 0.948 | 5016 | tags=29%, list=15%, signal=34% |
| KEGG_GLYCEROPHOSPHOLIPID_METABOLISM | KEGG_GLYCEROPHOSPHOLIPID_METABOLISM | | 77 | 0.363904 | 1.253242 | 0.165692 | 0.324024 | 0.957 | 5752 | tags=30%, list=18%, signal=36% |
| KEGG_EPITHELIAL_CELL_SIGNALING_IN_HELICOBACTER_PYLORI_INFECTION | KEGG_EPITHELIAL_CELL_SIGNALING_IN_HELICOBACTER_PYLORI_INFECTION | | 68 | 0.369988 | 1.251727 | 0.225806 | 0.320497 | 0.957 | 5892 | tags=37%, list=18%, signal=45% |
| KEGG_GLYCOSAMINOGLYCAN_BIOSYNTHESIS_HEPARAN_SULFATE | KEGG_GLYCOSAMINOGLYCAN_BIOSYNTHESIS_HEPARAN_SULFATE | | 26 | 0.416165 | 1.234632 | 0.185263 | 0.336967 | 0.962 | 5746 | tags=38%, list=18%, signal=47% |
| KEGG_PROGESTERONE_MEDIATED_OOCYTE_MATURATION | KEGG_PROGESTERONE_MEDIATED_OOCYTE_MATURATION | | 85 | 0.354616 | 1.226549 | 0.223141 | 0.340958 | 0.964 | 5955 | tags=33%, list=18%, signal=40% |
| KEGG_MAPK_SIGNALING_PATHWAY | KEGG_MAPK_SIGNALING_PATHWAY | | 266 | 0.328142 | 1.211609 | 0.215385 | 0.35636 | 0.967 | 7111 | tags=36%, list=22%, signal=46% |
| KEGG_ACUTE_MYELOID_LEUKEMIA | KEGG_ACUTE_MYELOID_LEUKEMIA | | 57 | 0.397573 | 1.191433 | 0.304904 | 0.375465 | 0.974 | 6009 | tags=33%, list=18%, signal=41% |
| KEGG_STARCH_AND_SUCROSE_METABOLISM | KEGG_STARCH_AND_SUCROSE_METABOLISM | | 52 | 0.393994 | 1.187142 | 0.262055 | 0.376651 | 0.977 | 3103 | tags=29%, list=9%, signal=32% |
| KEGG_O_GLYCAN_BIOSYNTHESIS | KEGG_O_GLYCAN_BIOSYNTHESIS | | 30 | 0.415818 | 1.175277 | 0.290258 | 0.386301 | 0.983 | 3970 | tags=30%, list=12%, signal=34% |
| KEGG_RNA_DEGRADATION | KEGG_RNA_DEGRADATION | | 59 | 0.368183 | 1.165543 | 0.317025 | 0.392605 | 0.983 | 9436 | tags=53%, list=29%, signal=74% |
| KEGG_APOPTOSIS | KEGG_APOPTOSIS | | 87 | 0.372535 | 1.158429 | 0.321212 | 0.396047 | 0.985 | 3344 | tags=23%, list=10%, signal=26% |
| KEGG_INSULIN_SIGNALING_PATHWAY | KEGG_INSULIN_SIGNALING_PATHWAY | | 137 | 0.294731 | 1.101911 | 0.342268 | 0.469645 | 0.99 | 6009 | tags=28%, list=18%, signal=35% |
| KEGG_ONE_CARBON_POOL_BY_FOLATE | KEGG_ONE_CARBON_POOL_BY_FOLATE | | 17 | 0.413232 | 1.093566 | 0.337255 | 0.475438 | 0.992 | 5123 | tags=35%, list=16%, signal=42% |
| KEGG_FC_GAMMA_R_MEDIATED_PHAGOCYTOSIS | KEGG_FC_GAMMA_R_MEDIATED_PHAGOCYTOSIS | | 96 | 0.343538 | 1.076437 | 0.414687 | 0.493816 | 0.995 | 5064 | tags=28%, list=15%, signal=33% |
| KEGG_DORSO_VENTRAL_AXIS_FORMATION | KEGG_DORSO_VENTRAL_AXIS_FORMATION | | 24 | 0.378565 | 1.065309 | 0.393004 | 0.503363 | 0.995 | 7639 | tags=42%, list=23%, signal=54% |
| KEGG_AMINO_SUGAR_AND_NUCLEOTIDE_SUGAR_METABOLISM | KEGG_AMINO_SUGAR_AND_NUCLEOTIDE_SUGAR_METABOLISM | | 43 | 0.339056 | 1.051741 | 0.420118 | 0.516651 | 0.996 | 9286 | tags=53%, list=28%, signal=75% |
| KEGG_NOD_LIKE_RECEPTOR_SIGNALING_PATHWAY | KEGG_NOD_LIKE_RECEPTOR_SIGNALING_PATHWAY | | 62 | 0.36201 | 1.014872 | 0.467909 | 0.564511 | 0.996 | 6987 | tags=34%, list=21%, signal=43% |
| KEGG_RIBOFLAVIN_METABOLISM | KEGG_RIBOFLAVIN_METABOLISM | | 15 | 0.351226 | 1.01219 | 0.437628 | 0.561628 | 0.996 | 5037 | tags=27%, list=15%, signal=32% |
| KEGG_FRUCTOSE_AND_MANNOSE_METABOLISM | KEGG_FRUCTOSE_AND_MANNOSE_METABOLISM | | 33 | 0.319329 | 0.974724 | 0.499029 | 0.614956 | 0.998 | 5168 | tags=30%, list=16%, signal=36% |
| KEGG_PENTOSE_AND_GLUCURONATE_INTERCONVERSIONS | KEGG_PENTOSE_AND_GLUCURONATE_INTERCONVERSIONS | | 28 | 0.365773 | 0.962195 | 0.505929 | 0.627377 | 0.998 | 3121 | tags=21%, list=10%, signal=24% |
| KEGG_B_CELL_RECEPTOR_SIGNALING_PATHWAY | KEGG_B_CELL_RECEPTOR_SIGNALING_PATHWAY | | 75 | 0.332144 | 0.932166 | 0.561587 | 0.668324 | 0.998 | 7057 | tags=37%, list=22%, signal=47% |
| KEGG_PORPHYRIN_AND_CHLOROPHYLL_METABOLISM | KEGG_PORPHYRIN_AND_CHLOROPHYLL_METABOLISM | | 40 | 0.313598 | 0.929476 | 0.555556 | 0.664514 | 0.998 | 6473 | tags=30%, list=20%, signal=37% |
| KEGG_MTOR_SIGNALING_PATHWAY | KEGG_MTOR_SIGNALING_PATHWAY | | 52 | 0.285006 | 0.908852 | 0.576017 | 0.692067 | 0.999 | 6979 | tags=35%, list=21%, signal=44% |
| KEGG_CARDIAC_MUSCLE_CONTRACTION | KEGG_CARDIAC_MUSCLE_CONTRACTION | | 78 | 0.246763 | 0.905727 | 0.574424 | 0.689617 | 0.999 | 2664 | tags=14%, list=8%, signal=15% |
| KEGG_STEROID_HORMONE_BIOSYNTHESIS | KEGG_STEROID_HORMONE_BIOSYNTHESIS | | 55 | 0.332571 | 0.901547 | 0.617155 | 0.68854 | 0.999 | 3370 | tags=18%, list=10%, signal=20% |
| KEGG_PURINE_METABOLISM | KEGG_PURINE_METABOLISM | | 154 | 0.227197 | 0.891534 | 0.603884 | 0.696797 | 0.999 | 5232 | tags=22%, list=16%, signal=26% |
| KEGG_SNARE_INTERACTIONS_IN_VESICULAR_TRANSPORT | KEGG_SNARE_INTERACTIONS_IN_VESICULAR_TRANSPORT | | 38 | 0.26292 | 0.883322 | 0.600406 | 0.701935 | 0.999 | 6849 | tags=34%, list=21%, signal=43% |
| KEGG_VIBRIO_CHOLERAE_INFECTION | KEGG_VIBRIO_CHOLERAE_INFECTION | | 54 | 0.234539 | 0.88147 | 0.633397 | 0.696935 | 0.999 | 5970 | tags=24%, list=18%, signal=29% |
| KEGG_GLYOXYLATE_AND_DICARBOXYLATE_METABOLISM | KEGG_GLYOXYLATE_AND_DICARBOXYLATE_METABOLISM | | 16 | 0.313286 | 0.875142 | 0.612717 | 0.699715 | 0.999 | 1959 | tags=13%, list=6%, signal=13% |
| KEGG_ETHER_LIPID_METABOLISM | KEGG_ETHER_LIPID_METABOLISM | | 33 | 0.29108 | 0.873426 | 0.647638 | 0.694625 | 0.999 | 2576 | tags=15%, list=8%, signal=16% |
| KEGG_TOLL_LIKE_RECEPTOR_SIGNALING_PATHWAY | KEGG_TOLL_LIKE_RECEPTOR_SIGNALING_PATHWAY | | 102 | 0.280041 | 0.862605 | 0.625263 | 0.703777 | 1 | 9958 | tags=48%, list=30%, signal=69% |
| KEGG_CELL_ADHESION_MOLECULES_CAMS | KEGG_CELL_ADHESION_MOLECULES_CAMS | | 131 | 0.306432 | 0.848931 | 0.623632 | 0.718435 | 1 | 3938 | tags=24%, list=12%, signal=28% |
| KEGG_NATURAL_KILLER_CELL_MEDIATED_CYTOTOXICITY | KEGG_NATURAL_KILLER_CELL_MEDIATED_CYTOTOXICITY | | 132 | 0.272664 | 0.847646 | 0.638298 | 0.712683 | 1 | 6273 | tags=30%, list=19%, signal=36% |
| KEGG_PHENYLALANINE_METABOLISM | KEGG_PHENYLALANINE_METABOLISM | | 18 | 0.293104 | 0.845665 | 0.684314 | 0.707585 | 1 | 1232 | tags=17%, list=4%, signal=17% |
| KEGG_GLYCEROLIPID_METABOLISM | KEGG_GLYCEROLIPID_METABOLISM | | 49 | 0.257763 | 0.834053 | 0.718876 | 0.717286 | 1 | 5419 | tags=22%, list=17%, signal=27% |
| KEGG_T_CELL_RECEPTOR_SIGNALING_PATHWAY | KEGG_T_CELL_RECEPTOR_SIGNALING_PATHWAY | | 108 | 0.279679 | 0.833387 | 0.641237 | 0.710789 | 1 | 7057 | tags=36%, list=22%, signal=46% |
| KEGG_INOSITOL_PHOSPHATE_METABOLISM | KEGG_INOSITOL_PHOSPHATE_METABOLISM | | 54 | 0.269835 | 0.828347 | 0.657315 | 0.711626 | 1 | 4550 | tags=20%, list=14%, signal=24% |
| KEGG_SPHINGOLIPID_METABOLISM | KEGG_SPHINGOLIPID_METABOLISM | | 39 | 0.26394 | 0.821479 | 0.678788 | 0.714824 | 1 | 7021 | tags=33%, list=21%, signal=42% |
| KEGG_HUNTINGTONS_DISEASE | KEGG_HUNTINGTONS_DISEASE | | 180 | 0.196522 | 0.813004 | 0.636191 | 0.721326 | 1 | 4059 | tags=11%, list=12%, signal=12% |
| KEGG_PHOSPHATIDYLINOSITOL_SIGNALING_SYSTEM | KEGG_PHOSPHATIDYLINOSITOL_SIGNALING_SYSTEM | | 76 | 0.261506 | 0.81123 | 0.700624 | 0.71643 | 1 | 3585 | tags=17%, list=11%, signal=19% |
| KEGG_HISTIDINE_METABOLISM | KEGG_HISTIDINE_METABOLISM | | 29 | 0.281212 | 0.80231 | 0.738866 | 0.723459 | 1 | 2883 | tags=21%, list=9%, signal=23% |
| KEGG_LEISHMANIA_INFECTION | KEGG_LEISHMANIA_INFECTION | | 69 | 0.306707 | 0.796871 | 0.655462 | 0.724422 | 1 | 7266 | tags=32%, list=22%, signal=41% |
| KEGG_ASCORBATE_AND_ALDARATE_METABOLISM | KEGG_ASCORBATE_AND_ALDARATE_METABOLISM | | 25 | 0.316018 | 0.78797 | 0.711155 | 0.730498 | 1 | 3019 | tags=20%, list=9%, signal=22% |
| KEGG_VASOPRESSIN_REGULATED_WATER_REABSORPTION | KEGG_VASOPRESSIN_REGULATED_WATER_REABSORPTION | | 44 | 0.226812 | 0.776634 | 0.728953 | 0.740419 | 1 | 9644 | tags=43%, list=29%, signal=61% |
| KEGG_ALZHEIMERS_DISEASE | KEGG_ALZHEIMERS_DISEASE | | 165 | 0.179497 | 0.760684 | 0.73062 | 0.757381 | 1 | 3008 | tags=10%, list=9%, signal=11% |
| KEGG_CYTOKINE_CYTOKINE_RECEPTOR_INTERACTION | KEGG_CYTOKINE_CYTOKINE_RECEPTOR_INTERACTION | | 263 | 0.259566 | 0.758941 | 0.739316 | 0.752117 | 1 | 3129 | tags=17%, list=10%, signal=19% |
| KEGG_JAK_STAT_SIGNALING_PATHWAY | KEGG_JAK_STAT_SIGNALING_PATHWAY | | 155 | 0.2367 | 0.752985 | 0.766949 | 0.752946 | 1 | 4254 | tags=19%, list=13%, signal=21% |
| KEGG_VIRAL_MYOCARDITIS | KEGG_VIRAL_MYOCARDITIS | | 68 | 0.252399 | 0.711302 | 0.749465 | 0.805964 | 1 | 8522 | tags=38%, list=26%, signal=52% |
| KEGG_CYTOSOLIC_DNA_SENSING_PATHWAY | KEGG_CYTOSOLIC_DNA_SENSING_PATHWAY | | 54 | 0.213921 | 0.681566 | 0.845382 | 0.838236 | 1 | 8651 | tags=31%, list=26%, signal=43% |
| KEGG_RNA_POLYMERASE | KEGG_RNA_POLYMERASE | | 29 | 0.220158 | 0.666153 | 0.788054 | 0.849827 | 1 | 11142 | tags=31%, list=34%, signal=47% |
| KEGG_CITRATE_CYCLE_TCA_CYCLE | KEGG_CITRATE_CYCLE_TCA_CYCLE | | 31 | 0.188951 | 0.58 | 0.876768 | 0.935236 | 1 | 4541 | tags=13%, list=14%, signal=15% |
| KEGG_OTHER_GLYCAN_DEGRADATION | KEGG_OTHER_GLYCAN_DEGRADATION | | 16 | 0.20301 | 0.570554 | 0.944334 | 0.934103 | 1 | 10939 | tags=50%, list=33%, signal=75% |
| KEGG_STEROID_BIOSYNTHESIS | KEGG_STEROID_BIOSYNTHESIS | | 17 | 0.232994 | 0.532735 | 0.927063 | 0.951816 | 1 | 10098 | tags=41%, list=31%, signal=60% |
